# Deep cardiac phenotyping by cardiovascular magnetic resonance reveals subclinical focal and diffuse myocardial injury in patients with psoriasis (PSOR-COR study)

**DOI:** 10.1007/s00392-024-02456-9

**Published:** 2024-05-16

**Authors:** Jan Gröschel, Leonhard Grassow, Edyta Blaszczyk, Kerstin Lommel, Georgios Kokolakis, Robert Sabat, Jeanette Schulz-Menger

**Affiliations:** 1 Charité – Universitätsmedizin Berlin, corporate member of Freie Universität Berlin and Humboldt-Universität Zu Berlin, ECRC Experimental and Clinical Research Center, Lindenberger Weg 80, 13125 Berlin, Germany; 2 Working Group On Cardiovascular Magnetic Resonance, Experimental and Clinical Research Center, a joint cooperation between Charité Medical Faculty and the Max-Delbrück Center for Molecular Medicine, Berlin, Germany; 3 DZHK (German Centre for Cardiovascular Research), Partner Site Berlin, Berlin, Germany; 4 Department of Dermatology and Allergology, HELIOS Hospital Berlin-Buch, Berlin, Germany; 5 Charité – Universitätsmedizin Berlin, corporate member of Freie Universität Berlin Und Humboldt-Universität Zu Berlin, Psoriasis Research and Treatment Center, Department of Dermatology, Venerology and Allergology & Institute of Medical Immunology, Berlin, Germany; 6 Department of Cardiology and Nephrology, HELIOS Hospital Berlin-Buch, Berlin, Germany

**Keywords:** Cardiovascular magnetic resonance, Psoriasis, Cardiovascular disease, Tissue characterization, Fibrosis, Inflammation

## Abstract

**Background:**

Psoriasis vulgaris (PV) is a chronic inflammatory disorder frequently associated with cardiovascular disease (CVD). This study aims to provide a prospective tissue characterization in patients with PV without major CVD using cardiovascular magnetic resonance (CMR).

**Methods:**

Patients with PV underwent laboratory assessment, a 12-lead and 24-h ECG, and a CMR exam at a 1.5-T scanner. Scan protocol included assessment of left (LV) and right (RV) ventricular function and strain analysis, native and post-contrast T1 mapping, T2 mapping and late gadolinium enhancement (LGE).

**Results:**

In total, 60 PV patients (median(IQR) age in years: 50.0 (36.0–60.8); 34 men (56.7%)) were recruited and compared to 40 healthy volunteers (age in years: 49.5 (37.3–57.8); 21 men (53.0%)). No differences were found regarding LV and RV function (*p* = 0.78 and *p* = 0.75). Global radial and circumferential strains were lower in patients (*p* < 0.001 and *p* < 0.001, respectively). PV had higher global T1 times (1001 (982–1026) ms vs. 991 (968–1005) ms; *p* = 0.01) and lower global T2 times (48 (47–49) ms vs. 50 (48–51) ms; *p* < 0.001); however, all values were within local reference ranges. Focal non-ischemic fibrosis was observed in 17 (28.3%) PV patients.

**Conclusion:**

Deep cardiac phenotyping by CMR revealed subclinical myocardial injury in patients with PV without major CVD, despite preserved LV and RV function. Diffuse and focal fibrosis might be the first detectable signs of adverse tissue remodeling leading to reduced circumferential and radial myocardial deformation. In the background of local and systemic immunomodulatory therapy, no signs of myocardial inflammation were detected. The exact impact of immunomodulatory therapies on the myocardium needs to be addressed in future studies.

**Study registration:**

ISRCTN71534700

**Graphical Abstract:**

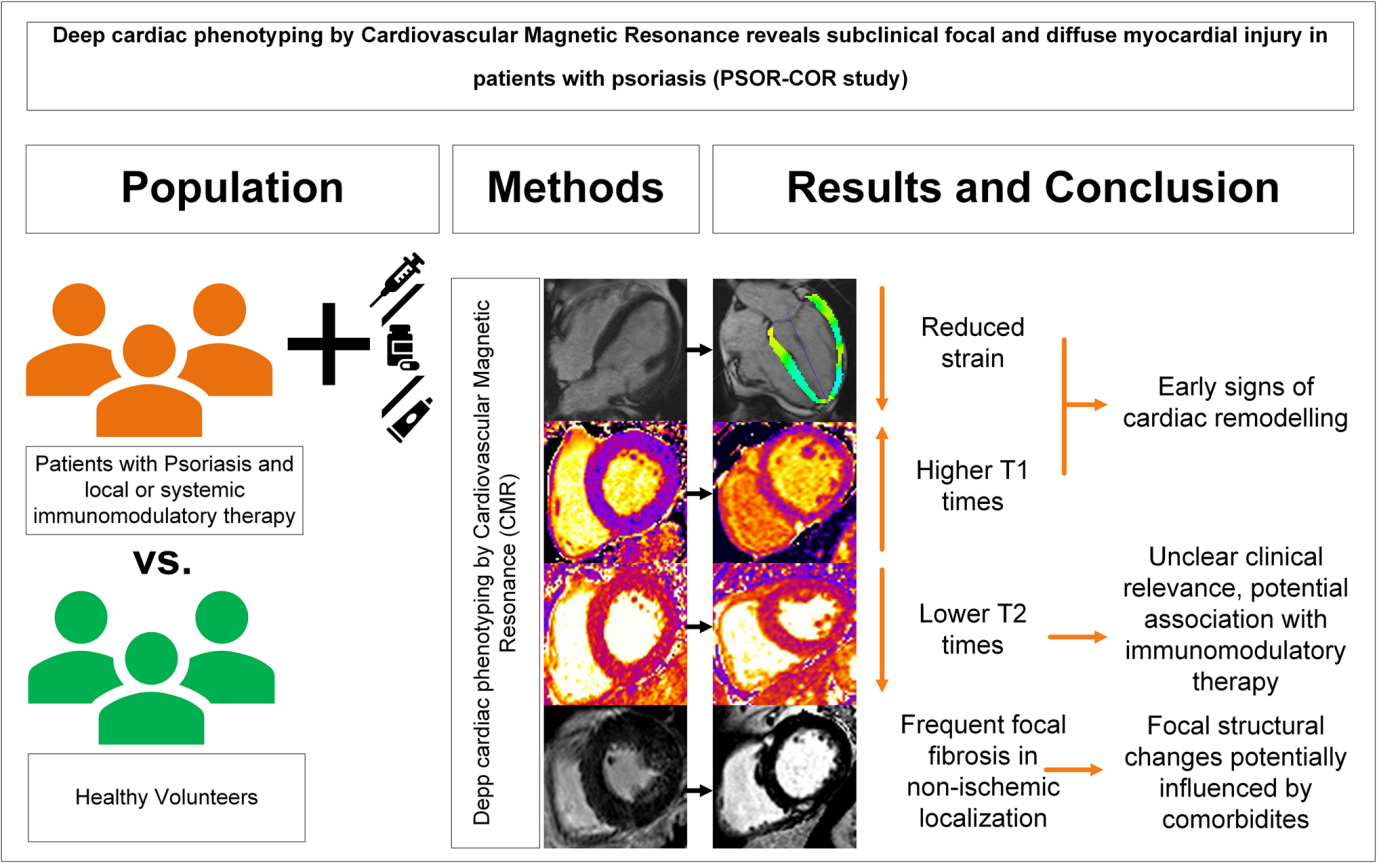

**Supplementary Information:**

The online version contains supplementary material available at 10.1007/s00392-024-02456-9.

## Introduction

**Fig. 1:**
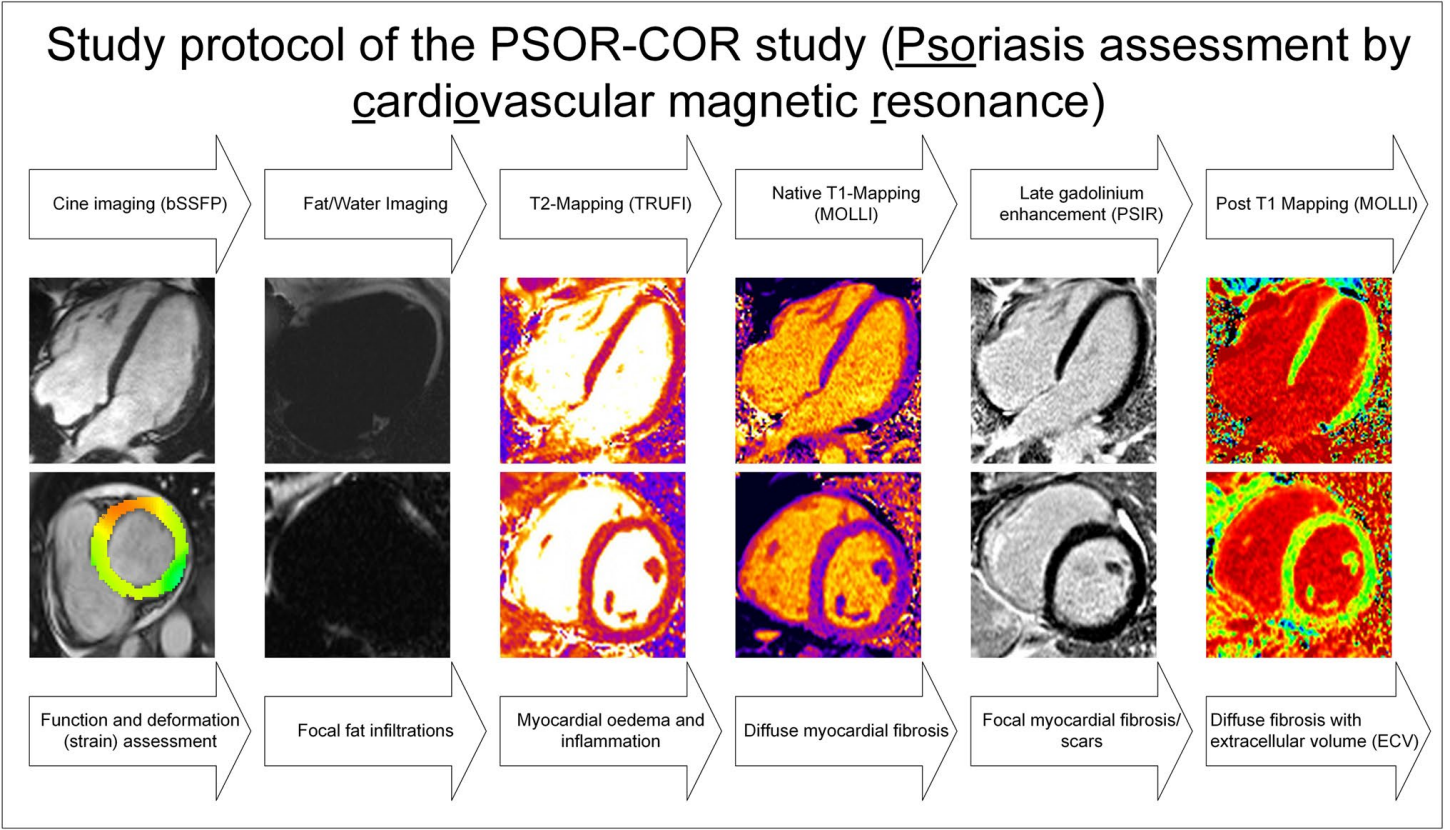
CMR protocol of the PCOR-COR study. Depicted are applied sequences (top) with the corresponding evaluation (bottom). bSSFP, balanced steady-state free precession; TRUFI, true fast imaging with steady-state free precession; MOLLI, modified Look-Locker imaging; PSIR, phase-sensitive inversion recovery

Psoriasis is a chronic systemic disorder primarily affecting the skin [1]. Multiple forms of psoriasis exist, the most prevalent being psoriasis vulgaris (PV) characterized by erythematous skin plaques with silvery-white scaling [2]. All forms have an immune system dysfunction in common, which leads to chronic inflammation [1, 3]. The pathophysiology has been extensively researched, and most aspects are well-understood. Current evidence suggests that interleukins, particularly interleukins (IL)-23 and IL-17, play a crucial role and are targeted by specific monoclonal antibodies [4]. These systemic therapies have been proven to be effective in reducing inflammation, skin alterations and disease burden [4, 5]. Although skin manifestations significantly impact patients’ psychological well-being, cardiovascular diseases (CVD) are the primary contributors to morbidity and mortality [6–8]. The interaction between PV and CVD is complex, with the main factors being the aforementioned inflammatory pathways and the high incidence of CVD risk factors [9–12]. Epidemiological cohort studies have shown that psoriasis is a major risk factor for developing coronary heart disease (CAD) [13, 14]. Despite recent evidence, guideline-directed therapies for CVD risk factors have only been partially implemented in this vulnerable cohort [15]. This contrasts with the widespread use of systemic cytokine-targeting therapies in PV. However, questions remain about the precise impact of PV and the effects of these new anti-inflammatory agents on the myocardium. Echocardiography studies involving PV patients have reported reduced global strain values [16, 17]. Furthermore, a prospective trial using coronary computed tomography found an association between left ventricular (LV) mass and non-calcific coronary artery disease in PV patients [18]. As CAD and finally myocardial infarction are the endpoints, the question remains whether changes in the myocardium, such as fibrosis and inflammation, might be detectable even earlier. In terms of imaging, cardiovascular magnetic resonance (CMR) can assess myocardial tissue characteristics [19, 20]. Parametric techniques, such as native and post-contrast T1 mapping for detecting diffuse myocardial fibrosis and T2 mapping for identifying myocardial edema, enable non-invasive differentiation of the myocardium [21]. Additionally, CMR can detect focal myocardial fibrosis through late gadolinium enhancement (LGE) imaging [22]. In this observational study, our aim was to provide in-depth myocardial characterization of patients with PV who have not experienced major cardiovascular events with a focus on fibrosis and inflammation, using the aforementioned techniques (see Fig. 1).

## Methods

### Study population

We conducted a prospective, exploratory observational study in patients with PV without major CVD, such as known CAD, cardiomyopathies and heart failure of any etiology, atrial fibrillation, severe valvular disease and COVID-19 infection within the last 3 months. Patients were recruited at outpatient dermatologic departments. Inclusion criteria were the following: diagnosed PV, age over 18 years and written informed consent. Exclusion criteria were pregnancy, breastfeeding, absolute contraindications for CMR and glomerular filtration rate < 30 ml/m^2^. The local ethical committee approved the study (EA1/130/21). A healthy volunteer (HV) cohort from previous studies was age and gender matched to the PV cohort [23–25]. Healthy was defined as the absence of previous cardiac, pulmonary, renal, or systemic disorders.

Patients’ treatment was chosen by the dermatologist following current guidelines. Dosages of immunomodulatory agents were chosen based on body weight or as recommended fixed dosages by the manufacturer at the time of treatment.

### CMR protocol

All participants underwent a CMR at a 1.5-T scanner (Avanto^FIT^, Siemens Healthineers, Erlangen, Germany) with an 18-channel surface coil. Scan protocol included cine imaging with a balanced steady-state free precession (bSSFP) sequence in four long axis views including a two-, three-, four- and a right ventricular (RV)-view, as well as a short axis stack (SAX). Fat/water imaging was carried out with a multi-echo sequence as described previously [26]. Parametric mapping was acquired in three SAX slices in basal, midventricular and apical locations, based on the previously published 5-out-of-3 approach [27]. T1 mapping was carried out with a 5–3-3 modified Look-Locker inversion (MOLLI) sequence. For T2 mapping, a bSSFP-based sequence was used, with the same positioning as the T1 mapping. After the application of 0.2 mmol/kg bodyweight of contrast media (gadoteridol, Prohance®, Bracco Imaging, Konstanz, Germany), LGE images were acquired by a phase-sensitive inversion recovery in long axis as well as a SAX stack. Post-contrast T1 mapping for the analysis of synthetic extracellular volume (ECV) was carried out with a prototype MOLLI [28] (Fig. 1). Except not receiving contrast media, the scanner, scan protocol and sequences used in the HV cohort did not differ from the patients’ protocol.

### Additional tests

Previous medical history and physical exam were performed at the time point of inclusion. Dermatologic evaluation was performed according to current recommendations including dermatologic scores such as Psoriasis Area and Severity Index (PASI) and Dermatologic Life Quality Index (DLQI) [29, 30]. Based on the therapy form, PV patients were divided into mild PV (mPV), receiving only local therapy, and moderate/severe PV (sPV), receiving systemic immunomodulatory therapy, groups [30]. All patients received a blood pressure measurement, a 12-lead electrocardiogram (ECG) as well as a 24-h Holter ECG. On the day of the CMR, a comprehensive blood laboratory panel was drawn including cardiologic (high sensitivity troponin T: local cutoff > 14 ng/L and NT-pro-BNP local cutoff > 125 ng/L) and inflammatory biomarkers as well as a complete blood count.

### CMR analysis

All CMR scans were analyzed with dedicated software (CVI42, Calgary, Canada version 5.17.0). Analysis was carried out in accordance with current recommendations and standard operating procedures [31]. LV and RV function and mass were analyzed in the SAX cine images with the inclusion of papillary muscles to the myocardial mass. Cardiac strain analysis was carried out by feature tracking as recently published [32]. Global values for longitudinal (GLS), radial (GRS) and circumferential (GCS) strains were calculated. T1- and T2-maps were visually assessed for artefacts or incorrect motion correction and accordingly excluded if present. Apical slices were disregarded due to a thin myocardium with increased partial volume effect [24]. Additionally, segments with focal myocardial fibrosis seen on LGE were excluded from the final analysis. Endo- and epicardial contours were drawn in all slices with a 5-% offset. Local cutoff values were as follows: T1 > 1037 ms, T2 > 54 ms and ECV > 24%. Fat/water images were inspected visually for fatty infiltration [26]. LGE images were visually assessed for focal myocardial fibrosis and scars. Findings were quantified according to location and subtype. For intra- and interreader comparisons, 10 random cases were contoured twice.

### Post-hoc subgroup comparisons

Given the common presence of comorbidities which could potentially influence parametric mapping results, especially arterial hypertension and diabetes mellitus type I or II, post-hoc subgroup analysis for PV patient without these comorbidities and HV was carried out. In addition, we wanted to examine the effect of immunomodulatory therapy on myocardial changes, providing a subgroup analysis between matched mild PV patients and HV.

### Statistical analysis

Continuous variables are presented as mean ± standard deviation (SD) or median with interquartile range (IQR). Categorical variables are represented as total and percent. Normal distribution was assessed by the Shapiro–Wilk test. Comparisons between PV and HV as well as the subgroups for continuous variables were carried out with unpaired *T*-tests or Mann–Whitney U tests. Categorical variables were compared with the chi-square test or Fisher’s exact test. Correlation analysis was carried out with Pearson’s or Spearman’s correlation coefficient. A *p* value of < 0.05 was considered statistically significant. Statistical analysis was carried out with SPSS (SPSS Statistics Version 29.0.0, IBM, Armok, NY, USA). Figures were created with Microsoft Visio (Microsoft Corporation, Redmond, WA, USA).

## Results

### Study population

Between February 2022 and March 2023, a total of 64 PV patients were recruited. Four patients had to be excluded, two due to an acute COVID-19 infection, one due to a newly diagnosed left-to-right cardiac shunt and one due to a newly diagnosed apical hypertrophic cardiomyopathy. The remaining 60 patients had a median (IQR) age of 50.0 (36.0–60.8) years. Out of the 60 participants, 34 were men (56.7%). In comparison to the HV, PV had a higher body mass index (BMI) (*p* = 0.01) (Table 1).

**Table 1:** General characteristics

Parameter	Psoriasis (PV) (*N* = 60)	Healthy volunteers (HV) (*N* = 40)	*p*-value PV vs. HV	Mild psoriasis (mPV) (*N* = 24)	Moderate/severe psoriasis (sPV) (*N* = 36)	*p*-value mPV vs. sPV
Sex (female/male)	26/34	19/21	0.68^‡^	10/14	16/20	0.83^‡^
Age (years)	50.0 (36.0–60.8)	49.5 (37.0–57.8)	0.83*	51.5 (35.3–61.8)	49.5 (37.3–60.0)	0.96*
Height (cm)	173.0 (166.3–180.8)	175.5 (168.0–181.8)	0.41*	170.5 (166.5–177.8)	175.5 (166.3–182.0)	0.21*
Weight (kg)	80.5 (70.0–90.8)	77.1 (66.1–83.5)	0.06^†^	80.0 (72.3–88.6)	81.1 (66.5–94.0)	0.66^**†**^
BMI (kg/m^2^)	26.5 (23.5–30.1)	24.2 (21.8–27.0)	**0.01**^**†**^	26.8 (24.0–30.3)	26.3 (23.3–30.1)	0.62^**†**^
BSA (m^2^)	2.0 (1.8–2.1)	1.9 (1.8–2.1)	0.33*	2.0 (1.8–2.0)	2.0 (1.8–2.2)	0.45*
Systolic blood pressure (mmHg)	119.0 (109.3–130.0)	119 (114.0–137.0)	0.28^**†**^	120.5 (113.0–131.5)	118.5 (108.3–129.3)	0.47^**†**^
Diastolic blood pressure (mmHg)	71.5 (64.3–76.0)	76.0 (70.0–80.0)	**0.03**^**†**^	71.0 (67.3–87.5)	72.0 (62.3–76.8)	0.80^**†**^

Data provided as absolute and percent or median and interquartile range. *BMI* body mass index, *BSA* body surface area, *PASI* Psoriasis Area and Severity Index, *DLQI* Dermatologic Life Quality Index. *T-tests, ^†^Mann–Whitney U test, ^‡^chi-square test or Fisher’s exact test

When questioned, PV patients reported dyspnea 11/60 (18%), palpitations 13/60 (22%), chest pain 4/60 (7%) and dizziness 13/60 (22%). Four patients had slight lower limb oedema on physical exam (4/60 (7%)). There was no difference in symptoms for dyspnea (*p* = 0.71), palpitations (*p* = 0.16), chest pain (*p* = 1.00) and dizziness (*p* = 1.00) between LGE − and LGE + patients. Major comorbidities included arterial hypertension (18/60, 30%) and hyperlipidemia (45/60, 75%) (Table 2).

**Table 2:** Dermatologic and cardiologic assessment of the psoriasis cohort

Parameter	Psoriasis (PV) (*N* = 60)
Disease duration (years)	22.0 (8.8–34.5)
PASI	2.6 (1.2–4.8)
DLQI	2.0 (0.3–4.0)
Joint involvement	9/60 (15%)
Nail involvement	26/60 (43.3%)
Smoking	22/60 (37%)
Arterial hypertension	18/60 (30%)
Diabetes mellitus type I	1/60 (1.7%)
Diabetes mellitus type II	2/60 (3.3%)
Heart failure	0/60 (0%)
Hyperlipidemia	45/60 (75%)
Coronary artery disease	0/60 (0%)
Peripheral arterial disease	0/60 (0%)
Arrhythmias	0/60 (0%)
Thyroid disease	5/60 (8.3%)
Chronic kidney disease	7/60 (11.7%)
Moderate valvular heart disease	2/60 (3.3%)
Metabolic syndrome	10/60 (17%)
COVID-19 Infection	29/60 (48.3%)
Topical anti-psoriatic treatment	29/60 (48.3%)
Calcipotriol	4/60 (6.7%)
Dimethyl fumarate	3/60 (5.0%)
Methotrexate	4/60 (6.7%)
IL-23 antibodies	16/60 (27%)
IL-17 antibodies	6/60 (10%)
Anti-TNF-alpha antibodies	6/60 (10%)

Data provided as median with interquartile range or absolute and percent. *PASI* Psoriasis Area and Severity Index, *DLQI* Dermatologic Life Quality Index, *IL* interleukin, *TNF-alpha* tumor necrosis factor alpha

Subgroup comparisons of mPV (*N* = 24) and sPV (*N* = 36) showed no significant differences regarding general characteristics, except joint and nail involvement (more common in sPV), arterial hypertension and the use of antihypertensive therapy (more common in mPV) (supplementary Table 1). Details regarding the anti-psoriatic therapy as well as treatment for comorbidities can be found in the supplementary Table 2.

### Additional tests

12-lead ECG as well as 24-h Holter ECG showed no significant findings. Ventricular extrasystole burden was low, despite one patient showing 491 ventricular extrasystoles and 47 runs of ventricular bigeminy. Laboratory assessment revealed elevated low-density lipoprotein levels (> 116 mg/dL) in 34/60 (57%) patients. In the overall cohort, elevated inflammatory parameters were found for high-sensitivity C-reactive protein (cutoff > 5 mg/dL) in 6/60 (10%), IL-6 (cutoff > 7 ng/L) in 6/60 (10%) and tumor necrosis factor-alpha (cutoff > 8.4 pg/mL) in 10/60 (17%) (supplementary Table 3).

### CMR results

#### Psoriasis in comparison to healthy volunteers

Global LV and RV function was preserved in PV with no significant differences to the HV (Table 3). Global radial and circumferential strain values were significantly lower in PV in comparison to HV (Table 3).

**Table 3:** Cardiac function and tissue parameters

Parameter	Psoriasis (PV) (*N* = 60)	Healthy volunteers (HV) (*N* = 40)	*p*-value PV vs. HV
LVEDV (ml)	140.1 (122.8–170.9)	134.7 (113.5–158.1)	0.48^†^
LVEDV-Index-height (mL/m)	84.0 (71.2–95.2)	76.7 (68.7–89.5)	0.39^†^
LVEDV-Index-BSA (mL/m^2^)	73.4 (62.2–82.9)	72.1 (65.2–83.8)	0.96^†^
LVESV (mL)	52.9 (44.1–68.2)	49.1 (41.1–60.7)	0.47^†^
LVSV (mL)	89.4 (75.7–101.0)	87.7 (74.4–105.1)	0.65^†^
LVSV-Index-BSA (mL/m^2^)	44.6 (41.6–52.1)	43.9 (42.0–53.6)	0.81^†^
LVEF (%)	63.0 (59.1–66.1)	63.0 (59.8–66.3)	0.78^†^
LV mass (g)	81.4 (71.1–99.9)	85.5 (75.6–110.3)	0.20^†^
LV mass-Index-BSA (mg/m^2^)	42.2 (37.2–50.3)	46.7 (41.2–53.3)	**0.01**^†^
RVEF (%)	54.6 (50.5–57.5)	53.3 (50.1–58.1)	0.75*
RVEDV (mL)	151.2 (130.7–186.9)	157.5 (134.5–183.7)	0.53^†^
RVEDV-Index-BSA (mL/m^2^)	79.2 (68.3–93.2)	84.9 (76.0–97.7)	0.12^†^
RVSV (mL)	85.2 (70.2–101.9)	83.4 (73.4–100.3)	0.82^†^
RVSV-Index-BSA (mL/m^2^)	44.3 (36.4–51.2)	45.0 (39.9–52.3)	0.16*
LA (cm^2^)	22.0 (18.8–24.7)	21.1 (18.6–23.4)	0.32^†^
LA EF (%)	64.0 (57.7–68.3)	62.7 (59.2–69.2)	0.77^†^
LA-EDV-Index-BSA (mL/m)	34.3 (28.4–41.5)	33.2 (28.2–38.7)	0.49^†^
RA (cm^2^)	21.3 (18.9–24.7)	21.1 (19.5–24.2)	0.65*
RA EF (%)	48.9 (43.9–55.2)	51.3 (46.1–57.7)	0.38^†^
Global longitudinal strain (%)	− 17.2 (− 18.3–(− 15.5))	− 17.1 (− 19.0–(− 16.1))	0.23^†^
Global radial strain (%)	24.0 (21.5–27.6)	28.0 (24.1–31.1)	< 0.001*
Global circumferential strain (%)	− 15.6 (− 17.3–(− 14.6))	− 17.5 (− 18.6–(− 15.9))	< 0.001*
T1 global (ms)	1001 (982–1026)	991 (968–1005)	0.01*
T1 basal (ms)	1004 (985–1024)	992 (970–1010)	0.01*
T1 midventricular (ms)	998 (974–1025)	986 (958–1001)	0.01*
T2 global (ms)	48 (47–49)	50 (48–51)	< 0.001*
T2 basal (ms)	48 (47–50)	50 (48–51)	0.003*
T2 midventricular (ms)	48 (47–49)	50 (48–51)	< 0.001*

Data provided as absolute and percent or median and interquartile range. *LV* left ventricle, *EDV* end-diastolic volume, *BSA* body surface area, *ESV* end-systolic volume, *SV* stroke volume, *EF* ejection fraction, *RV* right ventricle, *LA* left atrium, *RA* right atrium, *T-tests, ^†^Mann–Whitney U test

**Fig. 2:**
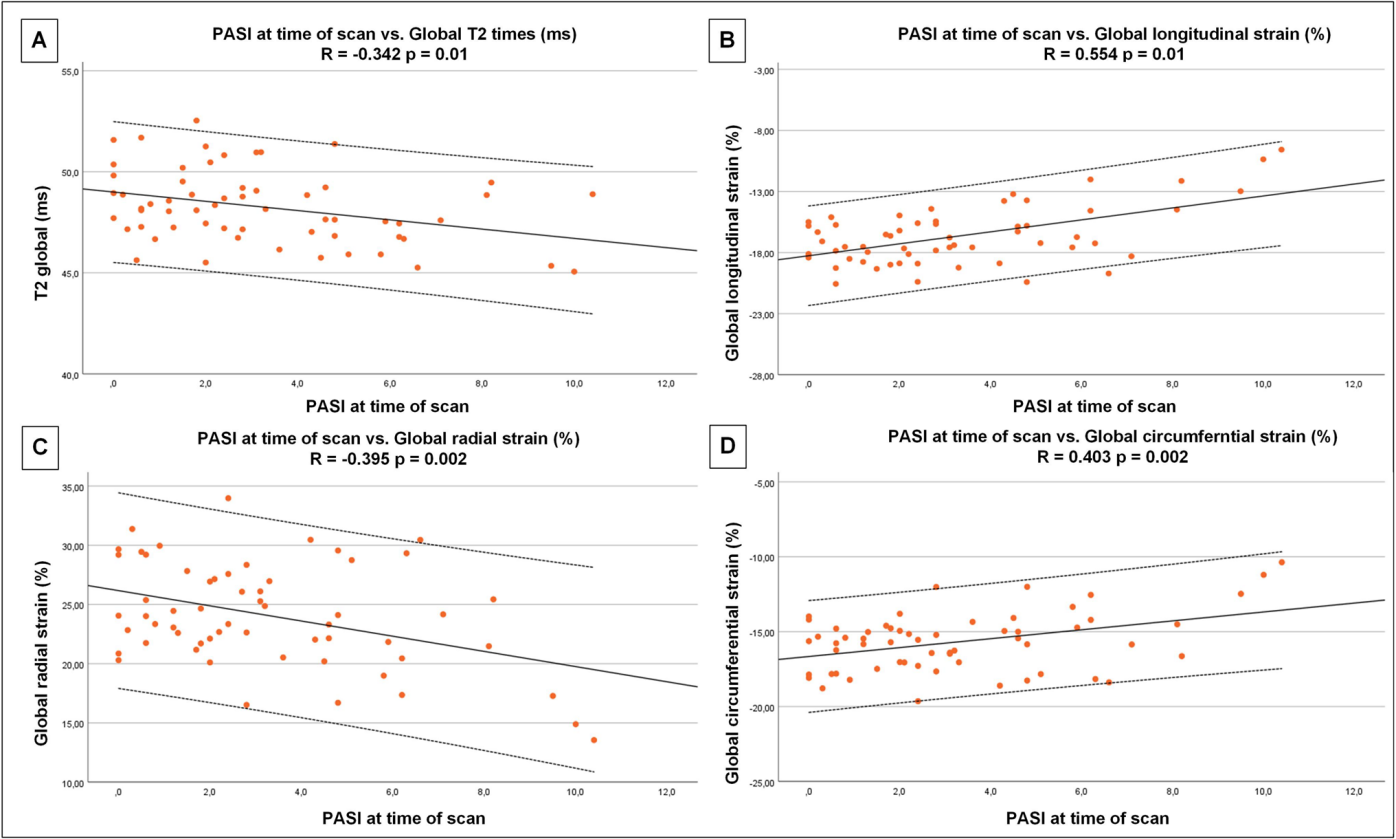
Correlation between PASI at the time of scan and T2 and left ventricular deformation indices. Significant correlations between PASI (Psoriasis Area and Severity Index) and T2 (**A**), global longitudinal (**B**), radial (**C**) and circumferential (**D**) strain

There were significant correlations between LV ejection fraction (LVEF) (*r* =  − 0.307; *p* = 0.02), GLS (*r* = 0.554; *p* = 0.01), GCS (*r* = 0.403; *p* = 0.002), GRS (*r* =  − 0.395; *p* = 0.002) and PASI (Fig. 2).

**Fig. 3:**
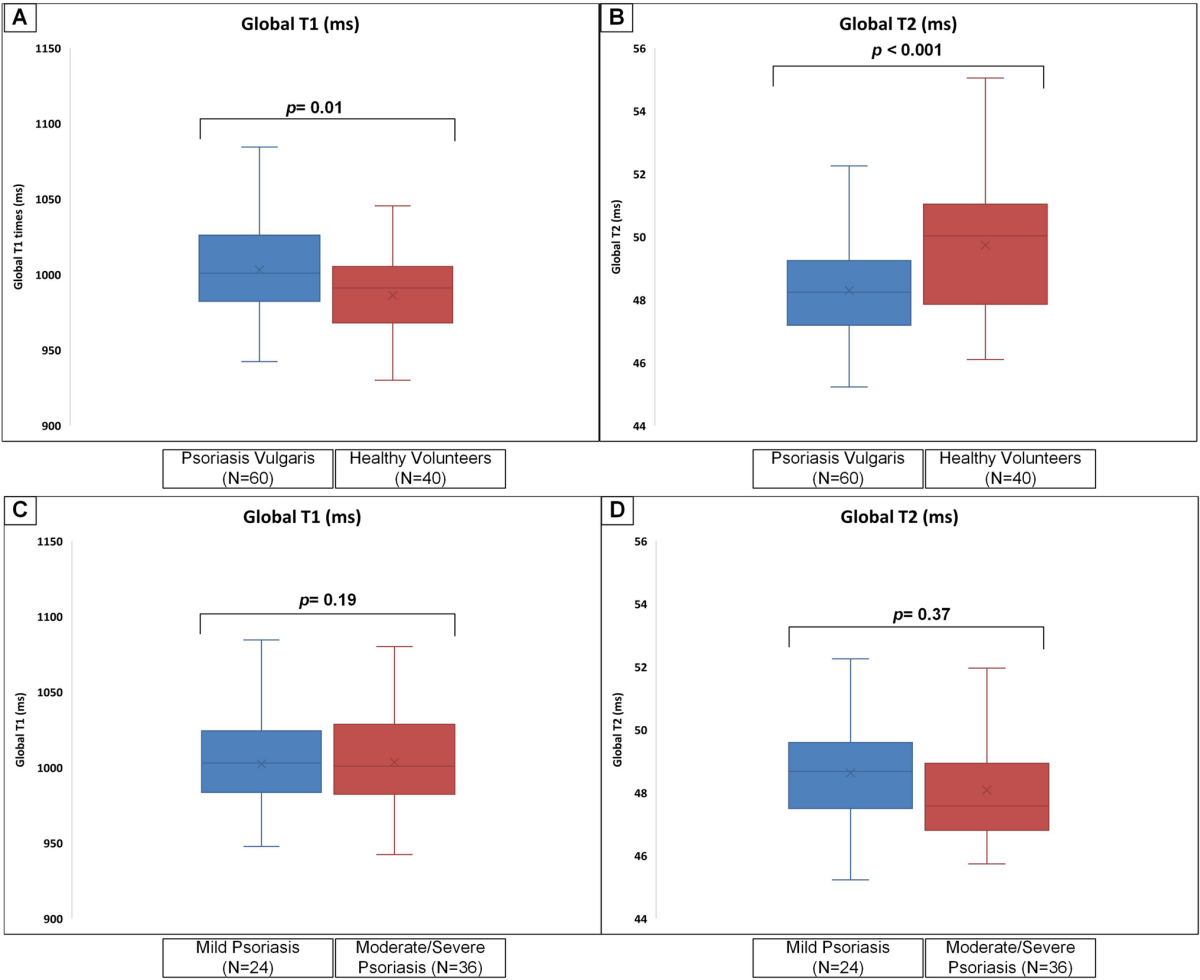
Comparison of native parametric myocardial tissue characterization between psoriasis patients and healthy volunteers and moderate and severe psoriasis. Boxplots representing the median (solid inside the box), interquartile range (box) and 1.5*interquartile range (whiskers) for T1 and T2 mapping for patients with psoriasis and healthy volunteers (**A** and **B**) as well as mild and moderate/severe psoriasis (**C** and **D**). Every value below or above 1.5*interquartile range is marked as an outlier. Significant differences were found between psoriasis and healthy volunteers

Patients with PV had higher global T1 times (*p* = 0.01), in basal (*p* = 0.01) and midventricular slices (*p* = 0.01) in comparison to the HV. T2 times were lower in the PV cohort. This was significant globally (*p* < 0.001) as well as in basal (*p* < 0.001) and in midventricular slices (*p* < 0.001) (Fig. 3).

Based on our centers reference ranges for parametric mapping, in the PV cohort, 7/60 (12%) patients had elevated global T1 times, 0/60 (0%) had elevated global T2 times and 16/60 (27%) had elevated global ECV values. Applying the same cutoffs in the HV group, one HV proband (1/40 (3%)) had elevated T1 times and two (2/40 (6%)) had elevated T2 times. This was neither statistically significant for T1 (*p* = 0.14) nor T2 (*p* = 0.2) in comparison to the HV.

**Fig. 4:**
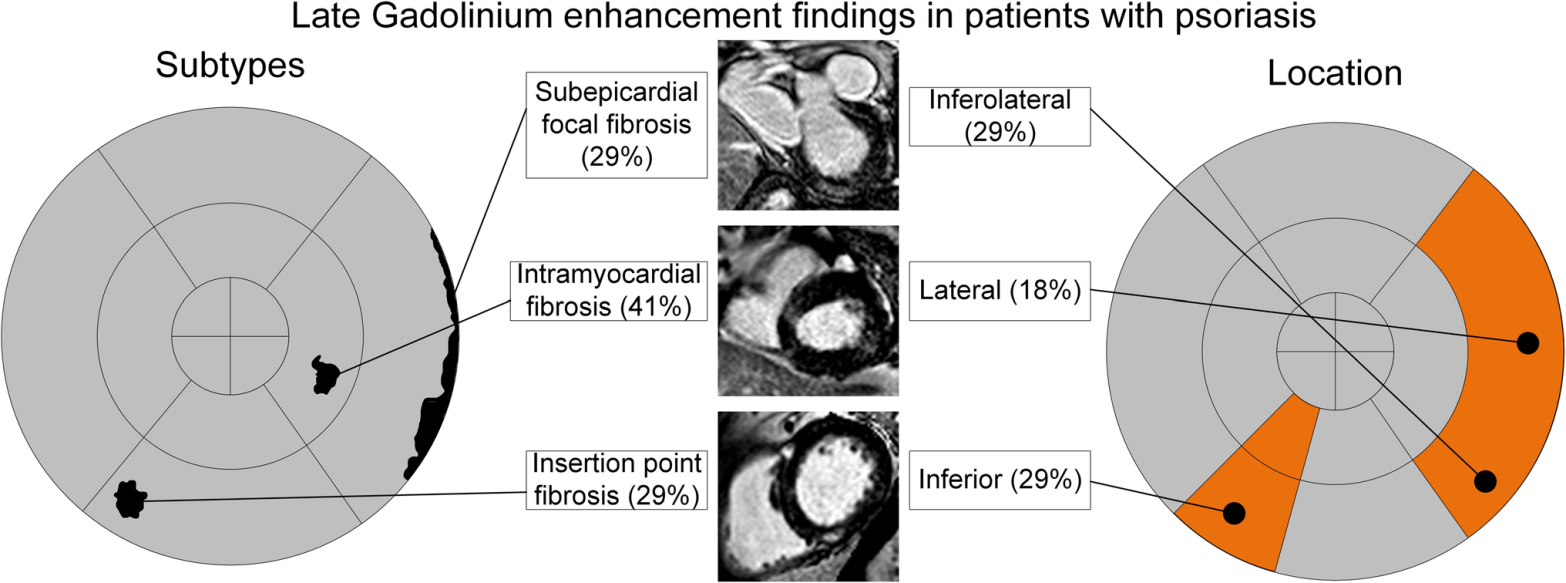
Overview of findings on myocardial tissue injury in patients with psoriasis. Bulls-eye representation of regions with focal fibrosis (right side of the figure). Most often the inferior and lateral walls were involved. Left side of the figure represents the affected myocardial layers, which were intramyocardial, subepicardial and insertion point fibrosis

There was a significant negative correlation between global T2 times and PASI (*r* =  − 0.342; *p* = 0.01). One patient had a small focal fatty infiltration in the apex of the LV. LGE was found in 17/60 (28%) of the PV patients. All in a non-ischemic pattern (supplementary Table 4, Fig. 4).

Most findings were in an intramyocardial location (7/17 (41%)), with the remaining equally split between subepicardial (5/17( 29%)) and the RV insertion point (5/17 (29%)). Basal inferior (5/17 (29%)), inferolateral (5/17 (29%)) and lateral (3/17 (18%)) regions were most often involved. Two focal myocardial fibrosis were found midventricular inferior (2/17 (12%)), one in the anteroseptal segment basally (1/17 (6%)) and one in the inferior segment apically (1/17 (6%)) (supplementary Table 4). Comparison of patients with LGE + (17/60; 28%) and LGE − (43/60; 72%) revealed no significant differences regarding myocardial deformation and T1 mapping (Supplementary Table 5). LGE + patients had lower LV-end-diastolic volume as well as lower left atrial ejection fraction in addition to higher T2 values in basal slices (supplementary Table 5). In comparison to HV, LGE − patients had a lower GRS (*p* < 0.001) and GCS (*p* < 0.001) (Supplementary Table 5). In addition, T1 times were higher (*p* = 0.02) and T2 times lower (*p* < 0.001) (Supplementary Table 5). Intra- and interreader comparisons can be found in supplementary Fig. 1.

In an additional subgroup comparison of PV patients without arterial hypertension and/or diabetes mellitus type I or II (*N* = 41/60), the main findings could be confirmed with PV patients having higher T1 times, lower T2 times and reduced GRS as well as GCS (supplementary Table 6).

#### Comparison of mild to moderate/severe psoriasis

The assessment of mPV and sPV regarding cardiac function, volumes and strain revealed no significant differences except higher RV end-diastolic volume. This, however, was not detectable anymore after adjusting for body surface area (supplementary Table 7). Myocardial tissue differentiation did not reveal any significant findings (Supplementary Table 7, Fig. 3). A further subgroup comparison between mPV and a matched HV subgroup (*N* = 24) showed lower GRS and GCS in the mPV group. However, in this subgroup comparison, no differences for T1 as well as T2 values were evident.

## Discussion

In this first prospective CMR study in patients with PV despite the absence of major CVD, we found evidence of subclinical myocardial injury. The main alterations detected in our study were signs of diffuse and focal myocardial fibrosis which were concordant with reduced deformation indices, potentially signifying subclinical myocardial tissue remodeling. Surprisingly, we did not detect signs of myocardial inflammation in the PV cohort. This might be related to a concomitant use of immunomodulatory therapies by PV patients.

Systemic disorders with a chronic inflammatory background have been frequently associated with CVD as the underlying pathophysiological pathways also affect the vasculature, especially coronary arteries on a macro- and microvascular level as well as the myocytes [33, 34]. Potential myocardial abnormalities could therefore be related to increased fibrotic tissue in a focal and diffuse distribution as well as evidence of inflammation. A brief report of a retrospective analysis of *N* = 49 patients with PV revealed that, in comparison to patients with rosacea and atopic dermatitis, 72% of the PV patients had an abnormal CMR, with the main findings including T2 and ECV elevations [35]. It should be of note, however, that the studied collective had a cardiologic indication for the scan. Other studies investigating patients with systemic disorders produced similar results. A study by Ntusi et al. included a cohort of *N* = 39 patients with rheumatic arthritis and no cardiologic symptoms [36]. The authors reported, despite a normal LV function, elevated T1 times, correlating with disease activity and in addition a high prevalence of focal myocardial findings (46%). Similar results were noted for systemic lupus erythematosus with elevated T1 findings [37]. A study by Mavrogeni et al. revealed an elevated T2-signal ratio in a mixed group with connective tissue disorders [38]. Our findings, especially the high prevalence of LGE and higher T1 times, expand these results. In addition, more than a quarter of our cohort had elevated global ECV times. However, a very important divergence is the non-elevated T2 times in our PV cohort. This came as a surprise as we expected higher values in accordance with a chronic inflammatory process [39, 40]. In addition, we found a negative correlation between the PASI and T2, with higher values in patients with a lower PASI. We can only speculate about the reasons for this finding. Thus, immunomodulatory suppression could be a crucial factor. Ntusi et al. reported reduced inflammatory markers and myocardial inflammation after anti-TNF-alpha treatment in a cohort with rheumatoid arthritis, ankylosing spondylitis and psoriatic arthritis [41]. Our findings might have a similar background as most of our PV patients were treated with potent anti-cytokine monoclonal antibodies. In fact, Makavos et al. followed-up patients with PV undergoing systemic immunomodulatory therapies, showing that specific anti-cytokine antibodies lead to a greater improvement in strain values as traditional anti-inflammatory agents [42]. The effect of systemic immunomodulatory might also have impacted the comparison between moderate and severe psoriasis as we did not detect a difference regarding tissue differentiation. The overall low disease activity is also signified by low PASI values in our cohort. Therefore, these findings need further research. Additional research and data in this area might be of value not only for PV but in regards of CVD, as there is evidence for a risk reduction while using systemic immunomodulatory therapies [43, 44].

The described findings in our PV cohort might be the first signs of cardiovascular remodeling. The reduced strain values underline the picture of a subclinical involvement in the PV cohort. In the recent American heart failure (HF) guidelines, a four-stage model was proposed, with stage A being patients at risk for HF [45]. Mentioned criteria include common CVD risk factors, such as obesity, metabolic syndrome and hypertension, as well as markers of early structural abnormalities such as reduced strain and left ventricular hypertrophy [45]. It should be apparent that patients with PV are exceptionally vulnerable, as CVD risk markers are common and, as demonstrated in this study, subclinical structural abnormalities are present. An echocardiographic study by Gorga et al. provided data showing a high prevalence of diastolic dysfunction in patients with PV [46]. There might be a related link to heart failure with preserved ejection fraction (HFpEF) [47, 48]. In conclusion, patients with PV seem to be at risk for HF, in specific HFpEF, and therefore, this connection should be used for developing targeted therapies acting at the molecular level to prevent or reduce the burden of HF. Valuable parameters to be tracked during such treatments could be GRS and GCS, based on the finding that only the radial deformation was impaired in our study.

However, the discussion should also include potential confounders and influencing factors in the analysis of the T1 values. Our PV cohort had a higher BMI. Both factors have been associated with elevated T1 values. Recently, Zhao et al. reported elevated markers of fibrosis as well as inflammation in a cohort of healthy obese probands in comparison to healthy normal-weight adults [49]. In the same study, an obese unhealthy group, based on the presence of more than one finding in accordance with a metabolic syndrome, had an even higher fibrotic burden [49]. CVD risk factors, and hence factors included in the definition of the metabolic syndrome, had a high prevalence among our PV cohort. The results of the subgroup comparison between PV patients without arterial hypertension and/or diabetes mellitus type I or II are however reassuring and provide evidence for a myocardial tissue alteration not solely being explainable by traditional CVD risk factors.

Future imaging studies addressing the association of PV and CVD should apply methods to detect microvascular changes, such as quantitative stress perfusion CMR or invasive measurements as recent reports noted a high burden of microvascular dysfunction in PV patients [50, 51].

Based on the LGE patterns found in the current study, relevant (unrecognized) myocardial infarctions are unlikely given the absence of subendocardial LGE. The subepicardial and intramyocardial fibrosis are described in the literature in association with myocardial inflammation; however, arterial hypertension can also cause such changes, especially intramyocardial ones [52]. RV insertion point fibrosis has been observed in healthy cohorts, therefore having an unclear clinical significance [53]. Unfortunately, even in the absence of subendocardial LGE, CAD cannot be certainly ruled out as no anatomical testing, such as by coronary computed tomography angiography, was not carried out. Given the recent evidence of large, randomized trials and the recommendation in guidelines, especially in a population with moderate pre-test probability, further research applying non-invasive anatomic testing is warranted [54–56].

## Limitations

The study is a single center. Additionally, no weight-matched healthy volunteer cohort was available. Due to ethical concerns, no contrast media was given in the healthy volunteer cohort preventing an analysis of LGE and ECV in this cohort. The scan protocol did not include stress perfusion imaging to detect myocardial ischemia as well as no anatomical testing for CAD; therefore, CAD cannot be excluded with certainty. In regards of subgroup comparisons, the compared numbers were rather small, therefore being potentially underpowered to observe statistically significant results. No follow-up data can be provided; therefore, no conclusions regarding the values of elevated T1 times as risk markers can be drawn. Lastly, laboratory assessment did not include high sensitivity CRP.

## Conclusion

Patients with psoriasis and no major cardiovascular history have signs of subclinical myocardial injury in the form of a reduced circumferential and radial myocardial strain as well as focal and diffuse fibrosis, potentially signifying a beginning myocardial remodeling process. Non-elevated T2 might be related to the use of systemic anti-cytokine therapies. Further studies are needed to evaluate the effect of immunomodulatory therapies on the myocardium.

## Supplementary Information

Below is the link to the electronic supplementary material.

Supplementary file1 (DOCX 16 KB)

Supplementary file2 (DOCX 16 KB)

Supplementary file3 (DOCX 16 KB)

Supplementary file4 (DOCX 20 KB)

Supplementary file5 (DOCX 14 KB)

Supplementary file6 (DOCX 349 KB)

Supplementary file7 (DOCX 18 KB)

Supplementary file8 (DOCX 16 KB)

## Data Availability

The datasets analyzed during the current study are not publicly available due to German laws but are available from the corresponding author on reasonable request.

## Abbreviations

PV: Psoriasis vulgaris
IL: Interleukin
CVD: Cardiovascular disease
CAD: Coronary artery disease
LV: Left ventricle
CMR: Cardiovascular magnetic resonance
LGE: Late gadolinium enhancement
HV: Healthy volunteers
bSSFP: Balanced steady-state free precession
RV: Right ventricle
SAX: Short axis
MOLLI: Modified Look-Locker inversion recovery sequence
ECV: Extracellular volume
PASI: Psoriasis Area and Severity Index
DLQI: Dermatologic Life Quality Index
mPV: Mild psoriasis vulgaris
sPV: Moderate/severe psoriasis vulgaris
ECG: Electrocardiogram
GLS: Global longitudinal strain
GRS: Global radial strain
GCS: Global circumferential strain
SD: Standard deviation
IQR: Interquartile range
BMI: Body mass index
LVEF: Left ventricular ejection fraction
HF: Heart failure
HFpEF: Heart failure with preserved ejection fraction

## References

[1] Boehncke WH, Schon MP (2015) Psoriasis. Lancet 386:983–994. 10.1016/S0140-6736(14)61909-7 26025581 10.1016/S0140-6736(14)61909-7

[2] Griffiths CEM, Armstrong AW, Gudjonsson JE, Barker JNWN (2021) Psoriasis. Lancet Lond Engl 397:1301–1315. 10.1016/S0140-6736(20)32549-6 10.1016/S0140-6736(20)32549-6 33812489

[3] Armstrong AW, Mehta MD, Schupp CW et al (2021) Psoriasis prevalence in adults in the United States. JAMA Dermatol 157:940–946. 10.1001/jamadermatol.2021.2007 34190957 10.1001/jamadermatol.2021.2007 PMC8246333

[4] Ghoreschi K, Balato A, Enerbäck C, Sabat R (2021) Therapeutics targeting the IL-23 and IL-17 pathway in psoriasis. Lancet Lond Engl 397:754–766. 10.1016/S0140-6736(21)00184-7 10.1016/S0140-6736(21)00184-7 33515492

[5] Armstrong AW, Read C (2020) Pathophysiology, clinical presentation, and treatment of psoriasis: a review. JAMA 323:1945–1960. 10.1001/jama.2020.4006 32427307 10.1001/jama.2020.4006

[6] Masson W, Lobo M, Molinero G (2020) Psoriasis and cardiovascular risk: a comprehensive review. Adv Ther 37:2017–2033. 10.1007/s12325-020-01346-6 32314303 10.1007/s12325-020-01346-6 PMC7467489

[7] Ahlehoff O, Gislason GH, Charlot M et al (2011) Psoriasis is associated with clinically significant cardiovascular risk: a Danish nationwide cohort study. J Intern Med 270:147–157. 10.1111/j.1365-2796.2010.02310.x 21114692 10.1111/j.1365-2796.2010.02310.x

[8] Garshick MS, Ward NL, Krueger JG, Berger JS (2021) Cardiovascular risk in patients with psoriasis: JACC review topic of the week. J Am Coll Cardiol 77:1670–1680. 10.1016/j.jacc.2021.02.009 33795041 10.1016/j.jacc.2021.02.009 PMC8168628

[9] Snekvik I, Nilsen TIL, Romundstad PR, Saunes M (2019) Metabolic syndrome and risk of incident psoriasis: prospective data from the HUNT study, Norway. Br J Dermatol 180:94–99. 10.1111/bjd.16885 29904911 10.1111/bjd.16885

[10] Qureshi AA, Choi HK, Setty AR, Curhan GC (2009) Psoriasis and the risk of diabetes and hypertension: a prospective study of US female nurses. Arch Dermatol 145:379–382. 10.1001/archdermatol.2009.48 19380659 10.1001/archdermatol.2009.48 PMC2849106

[11] Lonnberg AS, Skov L, Skytthe A et al (2016) Association of psoriasis with the risk for type 2 diabetes mellitus and obesity. JAMA Dermatol 152:761–767. 10.1001/jamadermatol.2015.6262 27120802 10.1001/jamadermatol.2015.6262

[12] Armstrong AW, Lin SW, Chambers CJ et al (2011) Psoriasis and hypertension severity: results from a case-control study. PLoS One 6:e18227. 10.1371/journal.pone.0018227 21479272 10.1371/journal.pone.0018227 PMC3066207

[13] Kaye JA, Li L, Jick SS (2008) Incidence of risk factors for myocardial infarction and other vascular diseases in patients with psoriasis. Br J Dermatol 159:895–902. 10.1111/j.1365-2133.2008.08707.x 18616778 10.1111/j.1365-2133.2008.08707.x

[14] Kimball AB, Guerin A, Latremouille-Viau D et al (2010) Coronary heart disease and stroke risk in patients with psoriasis: retrospective analysis. Am J Med 123:350–357. 10.1016/j.amjmed.2009.08.022 20362755 10.1016/j.amjmed.2009.08.022

[15] Kimball AB, Szapary P, Mrowietz U et al (2012) Underdiagnosis and undertreatment of cardiovascular risk factors in patients with moderate to severe psoriasis. J Am Acad Dermatol 67:76–85. 10.1016/j.jaad.2011.06.035 22018756 10.1016/j.jaad.2011.06.035

[16] Dattilo G, Imbalzano E, Casale M et al (2018) Psoriasis and cardiovascular risk: correlation between psoriasis and cardiovascular functional indices. Angiology 69:31–37. 10.1177/0003319717699329 29212353 10.1177/0003319717699329

[17] Bülbül Şen B, Ekiz Ö, Rifaioğlu EN et al (2016) Assessment of subclinical left ventricular dysfunction in patients with psoriasis by speckle tracking echocardiography: a Speckle Tracking Study. Int J Dermatol 55:158–164. 10.1111/ijd.12703 26104012 10.1111/ijd.12703

[18] Zhou W, Teklu M, Bui V et al (2021) The relationship between systemic inflammation and increased left ventricular mass is partly mediated by noncalcified coronary artery disease burden in psoriasis. Am J Prev Cardiol 7:100211. 10.1016/j.ajpc.2021.100211 34611643 10.1016/j.ajpc.2021.100211 PMC8387288

[19] Kawel-Boehm N, Maceira A, Valsangiacomo-Buechel ER et al (2015) Normal values for cardiovascular magnetic resonance in adults and children. J Cardiovasc Magn Reson 17:29. 10.1186/s12968-015-0111-7 25928314 10.1186/s12968-015-0111-7 PMC4403942

[20] Messroghli DR, Moon JC, Ferreira VM et al (2017) Clinical recommendations for cardiovascular magnetic resonance mapping of T1, T2, T2* and extracellular volume: a consensus statement by the Society for Cardiovascular Magnetic Resonance (SCMR) endorsed by the European Association for Cardiovascular Imaging (EACVI). J Cardiovasc Magn Reson Off J Soc Cardiovasc Magn Reson 19:75. 10.1186/s12968-017-0389-8 10.1186/s12968-017-0389-8 PMC5633041 28992817

[21] Ferreira VM, Schulz-Menger J, Holmvang G et al (2018) Cardiovascular magnetic resonance in nonischemic myocardial inflammation: expert recommendations. J Am Coll Cardiol 72:3158–3176. 10.1016/j.jacc.2018.09.072 30545455 10.1016/j.jacc.2018.09.072

[22] Fenski M, Grandy TH, Viezzer D et al (2022) Isotropic 3D compressed sensing (CS) based sequence is comparable to 2D-LGE in left ventricular scar quantification in different disease entities. Int J Cardiovasc Imaging. 10.1007/s10554-022-02571-6 35243574 10.1007/s10554-022-02571-6 PMC10509092

[23] Schmacht L, Traber J, Grieben U et al (2016) Cardiac involvement in myotonic dystrophy type 2 patients with preserved ejection fraction: detection by cardiovascular magnetic resonance. Circ Cardiovasc Imaging 9:e004615. 10.1161/CIRCIMAGING.115.004615 27363857 10.1161/CIRCIMAGING.115.004615

[24] von Knobelsdorff-Brenkenhoff F, J S, S D, et al (2017) Detection and monitoring of acute myocarditis applying quantitative cardiovascular magnetic resonance. Circ Cardiovasc Imaging 10. 10.1161/CIRCIMAGING.116.005242 10.1161/CIRCIMAGING.116.005242 28213448

[25] Birukov A, Wiesemann S, Golic M et al (2020) Myocardial evaluation of post-preeclamptic women by CMR: is early risk stratification possible? JACC Cardiovasc Imaging 13:1291–1293. 10.1016/j.jcmg.2020.01.005 32061559 10.1016/j.jcmg.2020.01.005

[26] Blaszczyk E, Lim C, Kellman P et al (2021) Progressive myocardial injury in myotonic dystrophy type II and facioscapulohumeral muscular dystrophy 1: a cardiovascular magnetic resonance follow-up study. J Cardiovasc Magn Reson Off J Soc Cardiovasc Magn Reson 23:130. 10.1186/s12968-021-00812-6 10.1186/s12968-021-00812-6 PMC8573966 34743704

[27] Messroghli DR, Bainbridge GJ, Alfakih K et al (2005) Assessment of regional left ventricular function: accuracy and reproducibility of positioning standard short-axis sections in cardiac MR imaging. Radiology 235:229–236. 10.1148/radiol.2351040249 15731374 10.1148/radiol.2351040249

[28] Gröschel J, Bhoyroo Y, Blaszczyk E et al (2022) Different impacts on the heart after COVID-19 infection and vaccination: insights from cardiovascular magnetic resonance. Front Cardiovasc Med 9:916922. 10.3389/fcvm.2022.916922 35911510 10.3389/fcvm.2022.916922 PMC9329612

[29] Langley RG, Ellis CN (2004) Evaluating psoriasis with Psoriasis Area and Severity Index, Psoriasis Global Assessment, and Lattice System Physician’s Global Assessment. J Am Acad Dermatol 51:563–569. 10.1016/j.jaad.2004.04.012 15389191 10.1016/j.jaad.2004.04.012

[30] Strober B, Ryan C, van de Kerkhof P et al (2020) Recategorization of psoriasis severity: Delphi consensus from the International Psoriasis Council. J Am Acad Dermatol 82:117–122. 10.1016/j.jaad.2019.08.026 31425723 10.1016/j.jaad.2019.08.026

[31] Schulz-Menger J, Bluemke DA, Bremerich J et al (2020) Standardized image interpretation and post-processing in cardiovascular magnetic resonance - 2020 update. J Cardiovasc Magn Reson 22:19. 10.1186/s12968-020-00610-6 32160925 10.1186/s12968-020-00610-6 PMC7066763

[32] Lim C, Blaszczyk E, Riazy L et al (2020) Quantification of myocardial strain assessed by cardiovascular magnetic resonance feature tracking in healthy subjects-influence of segmentation and analysis software. Eur Radiol. 10.1007/s00330-020-07539-5 33277669 10.1007/s00330-020-07539-5 PMC8128822

[33] Gargiulo P, Marsico F, Parente A et al (2014) Ischemic heart disease in systemic inflammatory diseases. An appraisal Int J Cardiol 170:286–290. 10.1016/j.ijcard.2013.11.048 24331863 10.1016/j.ijcard.2013.11.048

[34] Cheng C-Y, Baritussio A, Giordani AS et al (2022) Myocarditis in systemic immune-mediated diseases: prevalence, characteristics and prognosis A systematic review. Autoimmun Rev 21:103037. 10.1016/j.autrev.2022.103037 34995763 10.1016/j.autrev.2022.103037

[35] Goldenberg M, Reynolds M, Smart S et al (2020) A retrospective study of myocardial abnormalities detected on cardiac magnetic resonance imaging among patients with psoriasis compared to inflammatory skin disease controls. J Eur Acad Dermatol Venereol JEADV 34:e606–e608. 10.1111/jdv.16486 32299143 10.1111/jdv.16486 PMC7541559

[36] Ntusi NAB, Piechnik SK, Francis JM et al (2015) Diffuse myocardial fibrosis and inflammation in rheumatoid arthritis: insights from CMR T1 mapping. JACC Cardiovasc Imaging 8:526–536. 10.1016/j.jcmg.2014.12.025 25890584 10.1016/j.jcmg.2014.12.025

[37] Guo Q, Wu L-M, Wang Z et al (2018) Early detection of silent myocardial impairment in drug-naive patients with new-onset systemic lupus erythematosus: a three-center prospective study. Arthritis Rheumatol Hoboken NJ 70:2014–2024. 10.1002/art.40671 10.1002/art.40671 30070061

[38] Mavrogeni S, Sfikakis PP, Gialafos E et al (2014) Cardiac tissue characterization and the diagnostic value of cardiovascular magnetic resonance in systemic connective tissue diseases. Arthritis Care Res 66:104–112. 10.1002/acr.22181 10.1002/acr.22181 24106233

[39] Puntmann VO, Isted A, Hinojar R et al (2017) T1 and T2 mapping in recognition of early cardiac involvement in systemic sarcoidosis. Radiology 285:63–72. 10.1148/radiol.2017162732 28448233 10.1148/radiol.2017162732

[40] Zhang Y, Corona-Villalobos CP, Kiani AN et al (2015) Myocardial T2 mapping by cardiovascular magnetic resonance reveals subclinical myocardial inflammation in patients with systemic lupus erythematosus. Int J Cardiovasc Imaging 31:389–397. 10.1007/s10554-014-0560-3 25352245 10.1007/s10554-014-0560-3

[41] Ntusi NAB, Francis JM, Sever E et al (2018) Anti-TNF modulation reduces myocardial inflammation and improves cardiovascular function in systemic rheumatic diseases. Int J Cardiol 270:253–259. 10.1016/j.ijcard.2018.06.099 30017519 10.1016/j.ijcard.2018.06.099

[42] Makavos G, Ikonomidis I, Andreadou I et al (2020) Effects of interleukin 17A inhibition on myocardial deformation and vascular function in psoriasis. Can J Cardiol 36:100–111. 10.1016/j.cjca.2019.06.021 31606265 10.1016/j.cjca.2019.06.021

[43] Hjuler KF, Bottcher M, Vestergaard C et al (2016) Association between changes in coronary artery disease progression and treatment with biologic agents for severe psoriasis. JAMA Dermatol 152:1114–1121. 10.1001/jamadermatol.2016.1984 27385305 10.1001/jamadermatol.2016.1984

[44] Wu JJ, Joshi AA, Reddy SP et al (2018) Anti-inflammatory therapy with tumour necrosis factor inhibitors is associated with reduced risk of major adverse cardiovascular events in psoriasis. J Eur Acad Dermatol Venereol 32:1320–1326. 10.1111/jdv.14951 29573294 10.1111/jdv.14951

[45] Heidenreich PA, Bozkurt B, Aguilar D et al (2022) 2022 AHA/ACC/HFSA guideline for the management of heart failure: a report of the American College of Cardiology/American Heart Association Joint Committee on clinical practice guidelines. Circulation 145:e895–e1032. 10.1161/CIR.0000000000001063 35363499 10.1161/CIR.0000000000001063

[46] Gorga E, Scodro M, Valentini F et al (2018) Echocardiographic evaluation of diastolic dysfunction in young and healthy patients with psoriasis: a case-control study. Monaldi Arch Chest Dis Arch Monaldi Mal Torace 88:934. 10.4081/monaldi.2018.934 10.4081/monaldi.2018.934 30183154

[47] Pieske B, Tschöpe C, de Boer RA et al (2019) How to diagnose heart failure with preserved ejection fraction: the HFA-PEFF diagnostic algorithm: a consensus recommendation from the Heart Failure Association (HFA) of the European Society of Cardiology (ESC). Eur Heart J 40:3297–3317. 10.1093/eurheartj/ehz641 31504452 10.1093/eurheartj/ehz641

[48] Paulus WJ, Tschöpe C, Sanderson JE et al (2007) How to diagnose diastolic heart failure: a consensus statement on the diagnosis of heart failure with normal left ventricular ejection fraction by the Heart Failure and Echocardiography Associations of the European Society of Cardiology. Eur Heart J 28:2539–2550. 10.1093/eurheartj/ehm037 17428822 10.1093/eurheartj/ehm037

[49] Zhao H, Huang R, Jiang M, et al (2023) Myocardial tissue-level characteristics of adults with metabolically healthy obesity. JACC Cardiovasc Imaging S1936–878X(23)00095–5. 10.1016/j.jcmg.2023.01.022 10.1016/j.jcmg.2023.05.023 37558358

[50] Piaserico S, Papadavid E, Cecere A et al (2023) Coronary microvascular dysfunction in asymptomatic patients with severe psoriasis. J Invest Dermatol 143:1929-1936.e2. 10.1016/j.jid.2023.02.037 37739764 10.1016/j.jid.2023.02.037

[51] Piaserico S, Osto E, Famoso G et al (2019) Long-term prognostic value of coronary flow reserve in psoriasis patients. Atherosclerosis 289:57–63. 10.1016/j.atherosclerosis.2019.08.009 31476732 10.1016/j.atherosclerosis.2019.08.009

[52] Rudolph A, Abdel-Aty H, Bohl S et al (2009) Noninvasive detection of fibrosis applying contrast-enhanced cardiac magnetic resonance in different forms of left ventricular hypertrophy relation to remodeling. J Am Coll Cardiol 53:284–291. 10.1016/j.jacc.2008.08.064 19147047 10.1016/j.jacc.2008.08.064

[53] Joy G, Artico J, Kurdi H et al (2021) Prospective case-control study of cardiovascular abnormalities 6 months following mild COVID-19 in healthcare workers. JACC Cardiovasc Imaging 14:2155–2166. 10.1016/j.jcmg.2021.04.011 33975819 10.1016/j.jcmg.2021.04.011 PMC8105493

[54] Williams MC, Kwiecinski J, Doris M et al (2020) Low-attenuation noncalcified plaque on coronary computed tomography angiography predicts myocardial infarction: results from the multicenter SCOT-HEART trial (Scottish Computed Tomography of the HEART). Circulation 141:1452–1462. 10.1161/CIRCULATIONAHA.119.044720 32174130 10.1161/CIRCULATIONAHA.119.044720 PMC7195857

[55] DISCHARGE Trial Group, Maurovich-Horvat P, Bosserdt M et al (2022) CT or invasive coronary angiography in stable chest pain. N Engl J Med 386:1591–1602. 10.1056/NEJMoa2200963 35240010 10.1056/NEJMoa2200963

[56] Knuuti J, Wijns W, Saraste A et al (2020) 2019 ESC Guidelines for the diagnosis and management of chronic coronary syndromes. Eur Heart J 41:407–477. 10.1093/eurheartj/ehz425 31504439 10.1093/eurheartj/ehz425

