# Supplementary material for: Deep cardiac phenotyping by cardiovascular magnetic resonance reveals subclinical focal and diffuse myocardial injury in patients with psoriasis (PSOR-COR study)
Source: Clin Res Cardiol. 2024 May 16;114(9):1133–44. doi: 10.1007/s00392-024-02456-9 (PMC12408704; doi:10.1007/s00392-024-02456-9)
Supplement: Supplementary file 1 — Supplementary file1 (DOCX 16 KB) [file 392_2024_2456_MOESM1_ESM.docx]

Supplementary table1 General characteristics of mild and moderate/severe psoriasis

| Parameter | Mild Psoriasis (mPV) (N=24) | Moderate/severe Psoriasis (sPV) (N=36) | *p*-value mPV vs. sPV |
| --- | --- | --- | --- |
| Sex (female/male) | 10/14 | 16/20 | 0.83^‡^ |
| Age (years) | 51.5(35.3-61.8) | 49.5(37.3-60.0) | 0.96* |
| Height (cm) | 170.5(166.5-177.8) | 175.5(166.3-182.0) | 0.21* |
| Weight (kg) | 80.0(72.3-88.6) | 81.1(66.5-94.0) | 0.66**^†^** |
| BMI (kg/m^2^) | 26.8(24.0-30.3) | 26.3(23.3-30.1) | 0.62**^†^** |
| BSA (m^2^) | 2.0(1.8-2.0) | 2.0(1.8-2.2) | 0.45* |
| Systolic blood pressure (mmHg) | 120.5(113.0-131.5) | 118.5(108.3-129.3) | 0.47**^†^** |
| Diastolic blood pressure (mmHg) | 71.0(67.3-87.5) | 72.0(62.3 -76.8) | 0.80**^†^** |
| Disease Duration (years) | 17.5(6.8-31.5) | 26.0(11.3-35.5) | 0.66* |
| PASI | 2.8(1.9-5.0) | 2.2(0.6-4.8) | 0.14**^†^** |
| DLQI | 3.0(1.0-3.8) | 1.0(0.0-4.75) | 0.10**^†^** |
| Joint involvement | 0/24(0%) | 9/36(25%) | **0.01**^‡^ |
| Nail involvement | 5/24(20.8%) | 21/36(58.3%) | **0.01**^‡^ |
| Smoking | 7/24(29.2%) | 15/36(41.7%) | 0.32^‡^ |
| Arterial hypertension | 11/24(45.8%) | 7/36(19.4%) | **0.03**^‡^ |
| Diabetes Mellitus Type I | 0/24(0%) | 1/36(2.8%) | 1.0^‡^ |
| Diabetes Mellitus Type II | 1/24(4.2%) | 1/36(2.85) | 1.0^‡^ |
| Heart failure | 0/24(0%) | 0/36(0%) | 1.0^‡^ |
| Hyperlipidemia | 19/24(79.2%) | 26/36(72.2%) | 0.54^‡^ |
| Coronary artery disease | 0/24(0%) | 0/36(0%) | 1.0^‡^ |
| Peripheral arterial disease | 0/24(0%) | 0/36(0%) | 1.0^‡^ |
| Arrhythmias | 0/24(0%) | 0/36(0%) | 1.0^‡^ |
| Thyroid disease | 3/24(12.55) | 2/36(13.9%) | 0.38^‡^ |
| Chronic kidney disease | 2/24(8.3%) | 5/36(13.9%) | 1^‡^ |
| Moderate Valvular heart disease | 2/24(8.3%) | 0/36(0%) | 0.16^‡^ |
| Metabolic syndrome | 4/24(16.7%) | 6//36(16.7%) | 1^‡^ |
| COVID-19 Infection | 13/24(54.2%) | 16/36(44.4%) | 0.46^‡^ |

Data provided as absolute and percent or median and interquartile range. BMI=body mass index, BSA=body surface area, PASI=psoriasis area and severity index, DLQI= dermatologic life quality index, IL=interleukin, *T-tests, ^†^Mann-Whitney-U test, ^‡^Chi-square test or Fisher’s exact test.
