# Supplementary material for: Deep cardiac phenotyping by cardiovascular magnetic resonance reveals subclinical focal and diffuse myocardial injury in patients with psoriasis (PSOR-COR study)
Source: Clin Res Cardiol. 2024 May 16;114(9):1133–44. doi: 10.1007/s00392-024-02456-9 (PMC12408704; doi:10.1007/s00392-024-02456-9)
Supplement: Supplementary file 2 — Supplementary file2 (DOCX 16 KB) [file 392_2024_2456_MOESM2_ESM.docx]

Supplementary Table 2 Antipsoriatic and general treatments for comorbidities

| Parameter | Psoriasis (PV) (N=60) | Mild Psoriasis (mPV) (N=24) | Moderate/severe Psoriasis (sPV)(N=36) | p-value mPV vs. sPV |
| --- | --- | --- | --- | --- |
| Topical antipsoriatic treatment | 29/60 (48.3%) | 21/24 (87.5%) | 8/36 (22.2%) | **<0.001** |
| Calcipotriol | 4/60 (6.7%) | 1/24 (4.2%) | 3/36 (8.3%) | 0.64 |
| Dimethyl fumarate | 3/60 (5%) | 0/24 (0%) | 3/36 (8.3%) | 0.27 |
| Methotrexate | 4/60 (6.7%) | 0/24 (0%) | 4/36 (11.1%) | 0.14 |
| Ustekinumab | 8/60 (13.3%) | 0/24 (0%) | 8/36 (22.2%) | **0.02** |
| Etanercept | 1/60 (1.7%) | 0/24 (0%) | 1/36 (2.8%) | 1.0 |
| Secukinumab | 2/60 (3.3%) | 0/24 (0%) | 2/36 (5.6%) | 0.51 |
| Risankizumab | 5/60 (8.3%) | 0/24 (0%) | 5/36 (13.9%) | 0.08 |
| Ixekizumab | 3/60 (5%) | 0/24 (0%) | 3/36 (8.3%) | 0.27 |
| Guselkumab | 2/60 (3.3%) | 0/24 (0%) | 2/36 (5.6%) | 0.51 |
| Tildrakizumab | 1/60 (1.7%) | 0/24 (0%) | 1/36 (2.8%) | 1.0 |
| Infliximab | 2/60 (3.3%) | 0/24 (0%) | 2/36 (5.6%) | 0.51 |
| Adalimumab | 3/60 (5%) | 0/24 (0%) | 3/36 (8.3%) | 0.27 |
| Brodalumab | 1/60 (1.7%) | 0/24 (0%) | 1/36 (2.8%) | 1.0 |
| Aspirin | 1/60 (1.7%) | 1/24 (4.2%) | 0/36 (0%) | 0.40 |
| ACE/ARB | 12/60 (20%) | 8/24 (33.3%) | 4/36 (11.1%) | **0.049** |
| BB | 4/60 (6.7%) | 4/24 (16.7%) | 0/36 (0%) | **0.02** |
| MRA | 1/60 (1.7%) | 0/24 (0%) | 1/36 (2.8%) | 1.0 |
| Thiazide | 4/60 (6.7%) | 2/24 (8.3%) | 2/26 (5.6%) | 1.0 |
| CCB | 7/60 (11.7%) | 4/24 (16.7%) | 3/36 (8.3%) | 0.42 |
| Statin | 6/60 (10%) | 4/24 (16.7% | 2/36 (5.6%) | 0.21 |
| Ezetimibe | 1/60 (1.7%) | 1/24 (4.25) | 0/36 (0%) | 0.40 |
| Insulin | 2/60 (3.3%) | 0/24 (0%) | 2/36 (5.6%) | 0.51 |
| Metformin | 0/60 (0%) | 0/24 (0%) | 0/36 (0%) | 1.0 |

Data provided as absolute and percent. IL=interleukin, ACE=angiotensin converting enzyme inhibitors, ARB=angiotensin II receptor blockers, MRA=mineralocorticoid antagonists, CCB=calcium channel blockers. All Chi-square test or Fisher’s exact test.
