# Supplementary material for: Deep cardiac phenotyping by cardiovascular magnetic resonance reveals subclinical focal and diffuse myocardial injury in patients with psoriasis (PSOR-COR study)
Source: Clin Res Cardiol. 2024 May 16;114(9):1133–44. doi: 10.1007/s00392-024-02456-9 (PMC12408704; doi:10.1007/s00392-024-02456-9)
Supplement: Supplementary file 3 — Supplementary file3 (DOCX 16 KB) [file 392_2024_2456_MOESM3_ESM.docx]

Supplementary table 3 Laboratory and auxiliary tests in the psoriasis cohort

| Parameter | Psoriasis (N=60) | Mild Psoriasis (mPV) (N=24) | Moderate/severe Psoriasis (sPV) (N=36) | *p*-value mPV vs. sPV |
| --- | --- | --- | --- | --- |
| Creatinin µmol/l | 77 (70.3-93) | 78.5 (68.8-92.5) | 77.0 (70.5-99) | 0.41* |
| GFR % | 89.1 (72-104) | 92.0 (75.6-102.8) | 87.3 (65.0-104.0) | 0.45* |
| Triglycerides mg/dl | 147 (90.6-216) | 143.5 (92.5-274.0) | (151 (87.4-189.) | 0.46^†^ |
| Cholesterol mg/dl | 196 (166-221) | 180.0 (155.3-204.3) | 201.0 (178.0-229.0) | 0.06^†^ |
| LDL mg/dl | 122 (94.8-143) | 111.5 (89.1-132.8) | 128 (103.0-148.0) | 0.09^†^ |
| Lp(a) nmol/l | 11.9 (1.8-41) | 16.5 (7.5-67.8) | 11.0 (0.0-21.2) | 0.31^†^ |
| HDL mg/dl | 53.3 (44.1- 66.3) | 50.9 (44.4-62.3) | 55.5 (43.1-66.3) | 0.73^†^ |
| HbA1c % | 5.3 (5.0-5.5) | 5.2 (4.9-5.4) | 55.4 (5.1-5.6) | 0.12^†^ |
| Troponin T ng/l | 4.9 (3.3-6.9) | 6.0 (3.6-8.9) | 4.3 (3.2-5.8) | 0.09^†^ |
| CRP mg/l | 1.2 (0-3.1) | 1.6 (0.0-3.7) | 1.1 (0.0-2.4) | 0.47^†^ |
| NT-pro-BNP ng/l | 51.1 (23.8-104.8) | 53.2 (23.5-104.8) | 50.5 (25.2-104.5) | 0.84^†^ |
| Hb g/dl | 14.2 (13.3-15.1) | 14.7 (13.2-15.3) | 14.0 (13.3-15.0) | 0.98* |
| Erythrocytes Tpt/l | 4.6 (4.2-4.9) | 4.6 (4.3-5.0) | 4.6 (4.2-4.9) | 0.89* |
| Hct % | 41.2 (38.5-43.9) | 41.3 (39.1-44.9) | 41.1 (38.3-43.7) | 0.94* |
| MCV fl | 89.8 (87.9 -91.7) | 89.8 (87.9-92.3) | 89.9 (87.8-91.3) | 0.85* |
| MCH pg | 30.9 (30.0-31.8) | 31.0 (30.0-31.9) | 30.9 (29.9-31.8) | 0.83* |
| MCHC g/dl | 34.7 (33.8-35.2) | 34.7 (33.7-35.2) | 34.5 (33.8-35.2) | 0.75* |
| Thrombocytes Gpt/l | 251.5 (219-289.8) | 242.5 (194.3-312.3) | 253 (22.3-284.8) | 0.58* |
| Leukocytes Gpt/l | 7.6 (5.9-9.7) | 7.4 (5.8-9.6) | 7.7 (5.9-9.7) | 0.71^†^ |
| PCT µ/l | 0.03 (0.02-0.05) | 0.03 (0.02-0.05) | 0.03 (0.02-0,05) | 0.78^†^ |
| IL-6 ng/l | 2.3 (0.0-4.1) | 2.7 (1.6-5.0) | 2.0 (0.0-3.4) | 0.25^†^ |
| TNF-a pg/ml | 7.0 (6.1-9.3) | 7.0 (5.7-8.9) | 7.5 (6.2-9.7) | 0.47^†^ |

Data provided as median and interquartile range. GFR= glomerular filtration rate, LDL= low-density-lipoprotein, Lp(a) = lipoprotein (a), HDL= high-density lipoprotein, HbA1c= glycolated hemoglobin A, CRP= c-reactive protein, NT-pro-BNP= N-terminal prohormone of brain natriuretic peptide, Hb= hemoglobin, Hct= hematocrit, MCV= mean corpuscular volume, MCH= mean corpuscular hemoglobin, MCHC= mean corpuscular hemoglobin concentration, PCT= pro calcitonin, IL= interleukin, TNF-alpha= tumor necrosis factor alpha, *T-tests, ^†^Mann-Whitney-U test
