# Supplementary material for: Deep cardiac phenotyping by cardiovascular magnetic resonance reveals subclinical focal and diffuse myocardial injury in patients with psoriasis (PSOR-COR study)
Source: Clin Res Cardiol. 2024 May 16;114(9):1133–44. doi: 10.1007/s00392-024-02456-9 (PMC12408704; doi:10.1007/s00392-024-02456-9)
Supplement: Supplementary file 4 — Supplementary file4 (DOCX 20 KB) [file 392_2024_2456_MOESM4_ESM.docx]

Supplementary table 4 Cardiac function and tissue parameters for the healthy cohort, LGE- and LGE + psoriasis patients

| Parameter | Healthy volunteers (HV) (N=40) | LGE - Psoriasis (N=43) | LGE+ Psoriasis (N=17) | *p*-value HV vs. LGE- | *p*-value LGE- vs. LGE+ |
| --- | --- | --- | --- | --- | --- |
| LVEDV (ml) | 134.7(113.5-158.1) | 145.2 (123.2-176.2) | 136.6 (120.2-155.1) | 0.36^†^ | 0.45^†^ |
| LVEDV-Index-height (ml/m) | 76.7(68.7-89.5) | 76.8 (72.0-96.4) | 78.8 (70.9-86.9) | 0.26^†^ | 0.33^†^ |
| LVEDV-Index -BSA (ml/m^2^) | 72.1(65.2-83.8) | 76.8 (64.0-86.3) | 66.2 (61.5-74.5) | 0.56^†^ | 0.06^†^ |
| LVESV (ml) | 49.1(41.1-60.7) | 53.0 (44.2-69.7) | 50.1 (41.3-58.2) | 0.26^†^ | 0.20^†^ |
| LVSV (ml) | 87.7(74.4-105.1) | 90.1 (74.7-107.4) | 88.6 (81.6-98.5) | 0.70^†^ | 0.98^†^ |
| LVSV-Index -BSA (ml/m^2^) | 43.9(42.0-53.6) | 46.5 (41.3-52.9) | 43.8 (41.7-45.6) | 0.79^†^ | 0.15^†^ |
| LVEF (%) | 63.0(59.8-66.3) | 62.6 (59.0-65.9) | 65.8 (61.2-68.0) | 0.39* | 0.31* |
| LV mass (g) | 85.5(75.6-110.3) | 80.9 (67.8-98.9) | 82.6 (74.3-111.5) | 0.12^†^ | 0.32^†^ |
| LV mass-Index -BSA (mg/m^2^) | 46.7(41.2-53.3) | 42.5 (36.9-48.7) | 41.7 (37.2-53.7) | **0.02^†^** | 0.89^†^ |
| RVEF (%) | 53.3(50.1-58.1) | 53.3 (50.2-57.5) | 55.0 (51.0-58.1) | 0.92* | 0.55* |
| RVEDV (ml) | 157.5(134.5-183.7) | 160.0 (128.1-188.1) | 154.1 (138.0-181.2) | 0.52^†^ | 0.90^†^ |
| RVEDV-Index -BSA (ml/m^2^) | 84.9(76.0-97.7) | 80.7 (68.5-93.9) | 75.2 (68.1-90.1) | 0.28^†^ | 0.35^†^ |
| RVSV (ml) | 83.4(73.4-100.3) | 85.1 (66.5-102.6) | 85.4 (78.4-101.7) | 0.60^†^ | 0.68^†^ |
| RVSV-Index -BSA (ml/m^2^) | 45.0(39.9-52.3) | 44.7 (35.9-51.4) | 43.6 (37.0-49.5) | 0.28* | 0.57* |
| LA (cm^2^) | 21.1(18.6-23.4) | 21.7 (18.5-24.9) | 23.6 (19.4-25.9) | 0.95* | 0.16* |
| LA EF (%) | 62.7(59.2-69.2) | 64.3 (60.0-68.3) | 57.3 (52.4-66.5) | 0.63* | **0.046*** |
| LA-EDV-Index-BSA (ml/m) | 33.2(28.2-38.7) | 33.7 (27.9-41.3) | 35.2 (28.4-42.6) | 0.71^†^ | 0.50^†^ |
| RA (cm^2^) | 21.1(19.5-24.2) | 21.1 (18.6-24.3) | 23.4 (19.8-24.9) | 0.84* | 0.58* |
| RA EF (%) | 51.3(46.1-57.7) | 49.1 (44.0-56.3) | 48.7 (42.6 -54.8) | 0.52^†^ | 0.55^†^ |
| Global longitudinal Strain (%) | -17.1(-19.0-(-16.1) | -17.2 (-18.5-(-15.5) | 17.0 (-17.9-(-14.2) | 0.38^†^ | 0.46^†^ |
| Global radial Strain (%) | 28.0(24.1-31.1) | 24.0 (21.5-28.8) | 23.7 (20.9-26.7) | **0.002*** | 0.58* |
| Global circumferential Strain (%) | -17.5(-18.6-(-15.9) | -15.6 (-17.8 -(-14.6) | -15.6 (-16.9-(-14.4) | **0.002*** | 0.62* |
| T1 global (ms) | 991(968-1005) | 1001.3 (982.0-1025.4) | 993.6 (984.7-1030.1) | **0.02*** | 0.50* |
| T1 basal (ms) | 992(970-1010) | 1002.3 (983.1-1021.7) | 1005.6 (989.1-1030.4) | **0.03*** | 0.23* |
| T1 midventricular (ms) | 986(958-1001) | 998.3 (972.7-1021.9) | 997.3 (974.6-1029.7) | **0.01*** | 0.76* |
| T2 global (ms) | 50.0(47.9-51.0) | 47.9 (46.9-48.7) | 49.0 (47.9-50.4) | **<0.001*** | 0.12* |
| T2 basal (ms) | 49.9(48.1-50.8) | 48.2 (47.1-48.9) | 49.3 (47.4-50.3) | **<0.001*** | **0.03*** |
| T2 midventricular (ms) | 50.4(47.6-51.3) | 48.1 (46.6-48.6) | 48.3 (46.9-50.1) | **<0.001*** | 0.36* |
| ECV global (%) | n.a | 23.2 (21.2-24.2) | 23.1 (21.9-24.0) | n.a. | 0.76* |
| ECV basal (%) | n.a. | 22.6 (20.9-24.0) | 22.4 (21,7-23.8) | n.a. | 0.89* |
| ECV midventricular (%) | n.a. | 23.7 (21.4-24.6) | 22.9 (22.0-25.1) | n.a. | 0.81* |

Data provided as absolute and percent or median and interquartile range. LV=left ventricle, EDV=end-diastolic volume, BSA=body surface area, ESV=end-systolic volume, SV=stroke volume, EF=ejection fraction, RV=right ventricle, LA=left atrium, RA=right atrium, ECV=extracellular volume, LGE=late gadolinium enhancement. *T-tests, ^†^Mann-Whitney-U test, ^‡^Chi-square test or Fisher’s exact test.
