# Supplementary material for: Deep cardiac phenotyping by cardiovascular magnetic resonance reveals subclinical focal and diffuse myocardial injury in patients with psoriasis (PSOR-COR study)
Source: Clin Res Cardiol. 2024 May 16;114(9):1133–44. doi: 10.1007/s00392-024-02456-9 (PMC12408704; doi:10.1007/s00392-024-02456-9)
Supplement: Supplementary file 5 — Supplementary file5 (DOCX 14 KB) [file 392_2024_2456_MOESM5_ESM.docx]

Supplementary table 5. Extracellular volume and late gadolinium enhancement findings in the psoriasis cohort

| Parameter | Psoriasis (PV) (N=60) | Mild Psoriasis (mPV) (N=24) | Moderate/severe Psoriasis (sPV) (N=36) | *p*-value mPV vs. sPV |
| --- | --- | --- | --- | --- |
| ECV global (%) | 23.1 (21.3-24.1) | 22.9 (21.5-23.6) | 23.4 (21.2-24.2) | 0.72* |
| ECV basal (%) | 22.6 (21.0-24.0) | 22.5 (21.6-24.0) | 22.6 (20.9-24.0) | 0.94* |
| ECV midventricular (%) | 23.3 (21.5-24.7) | 22.8 (21.5-24.0) | 23.8 (21.5-25.0) | 0.54* |
| LGE present | 17/60 (28.3%) | 9/24 (37.5%) | 8/36 (22.2%) | 0.20^‡^ |
| LGE ischemic | 0/17 (0%) | 0/9 (0%) | 0/8 (0%) | 1^‡^ |
| LGE non-ischemic | 17/17 (100%) | 9/9 (100%) | 8/8 (100%) | 1^‡^ |
| LGE subepicardial | 5/17 (29%) | 3/9 (33%) | 2/8 (25%) | 0.71^‡^ |
| LGE intramyocardial | 7/17 (41%) | 4/9 (44%) | 3/8 (37.5%) | 0.77^‡^ |
| LGE RV insertionpoint | 5/17 (29%) | 2/9 (22%) | 3/8 (37.5%) | 0.49^‡^ |

ECV=extracellular volume, LGE=late gadolinium enhancement. *T-tests, ^†^Mann-Whitney-U test, ^‡^Chi-square test or Fisher’s exact test.
