# Supplementary material for: Deep cardiac phenotyping by cardiovascular magnetic resonance reveals subclinical focal and diffuse myocardial injury in patients with psoriasis (PSOR-COR study)
Source: Clin Res Cardiol. 2024 May 16;114(9):1133–44. doi: 10.1007/s00392-024-02456-9 (PMC12408704; doi:10.1007/s00392-024-02456-9)
Supplement: Supplementary file 6 — Supplementary file6 (DOCX 349 KB) [file 392_2024_2456_MOESM6_ESM.docx]

Supplementary figure 1 Bland-Altman plots for intra- and interreader comparisons


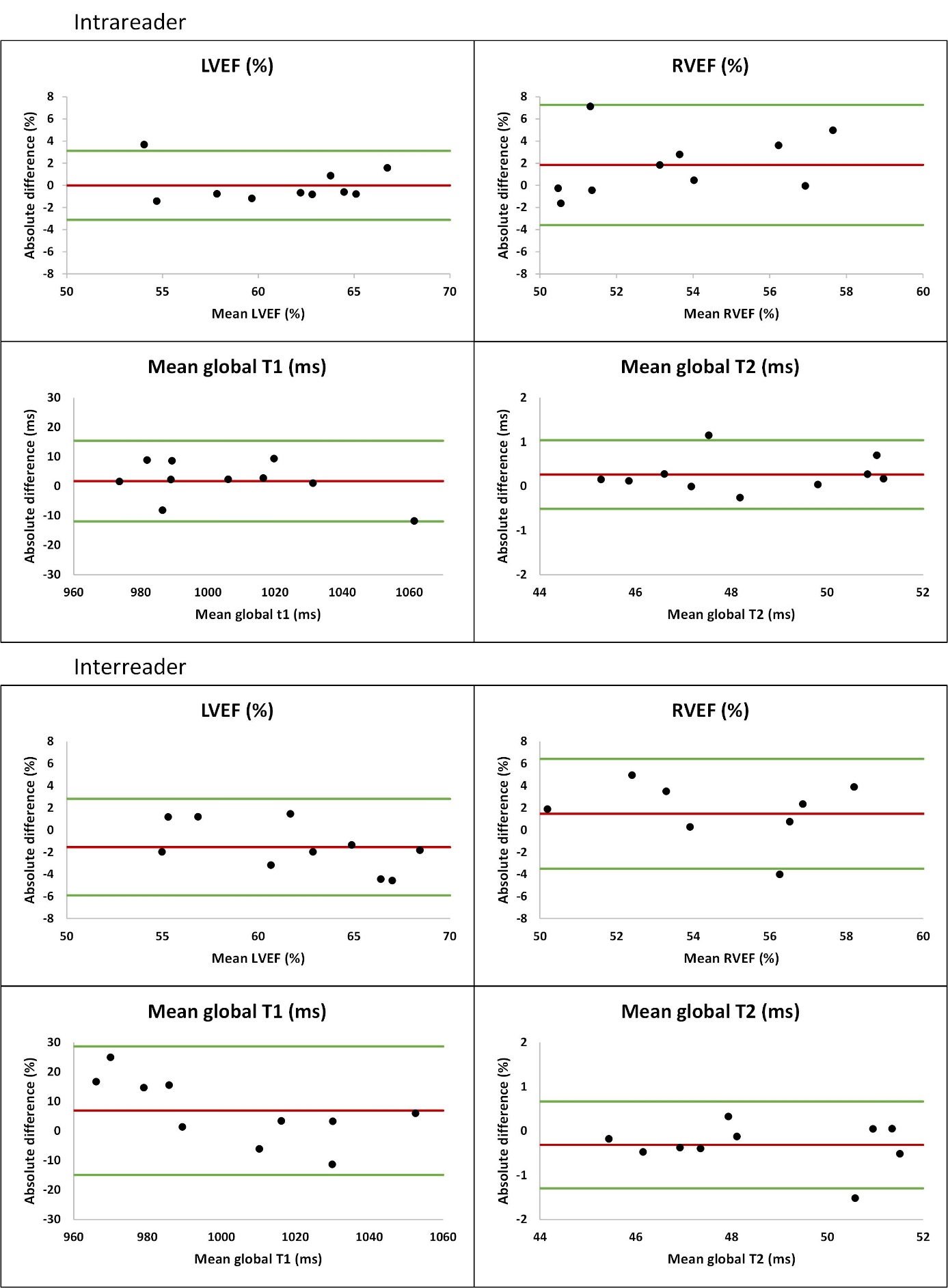


Bland-Altman analysis of intraobserver reproducibility of left ventricular ejection fraction, right ventricular ejection, global native T1 and global T2 values. Interobserver reproducibility of left ventricular ejection fraction, right ventricular ejection, global native T1 and global T2 values. Red line indicates mean difference; green lines indicate 95%-limits of agreement.
