# Supplementary material for: Deep cardiac phenotyping by cardiovascular magnetic resonance reveals subclinical focal and diffuse myocardial injury in patients with psoriasis (PSOR-COR study)
Source: Clin Res Cardiol. 2024 May 16;114(9):1133–44. doi: 10.1007/s00392-024-02456-9 (PMC12408704; doi:10.1007/s00392-024-02456-9)
Supplement: Supplementary file 7 — Supplementary file7 (DOCX 18 KB) [file 392_2024_2456_MOESM7_ESM.docx]

Supplementary table 6 Cardiac function and tissue parameters for the psoriasis cohort without arterial hypertension and diabetes mellitus

| Parameter | Psoriasis without aHTN and DM (N=42) | Healthy volunteers (HV) (N=40) | *p*-value PV vs. HV |
| --- | --- | --- | --- |
| LVEDV (ml) | 141.8(126.6-174.7) | 134.7(113.5-158.1) | 0.23^†^ |
| LVEDV-Index-height (ml/m) | 84.3(75.5-96.2) | 76.7(68.7-89.5) | 0.19^†^ |
| LVEDV-Index -BSA (ml/m^2^) | 75.6(64.4-87.9) | 72.1(65.2-83.8) | 0.55^†^ |
| LVESV (ml) | 54.7(44.8-67.7) | 49.1(41.1-60.7) | 0.24^†^ |
| LVSV (ml) | 90.4(78.7-105.5) | 87.7(74.4-105.1) | 0.43^†^ |
| LVSV-Index -BSA (ml/m^2^) | 46.1(42.1-53.7) | 43.9(42.0-53.6) | 0.68^†^ |
| LVEF (%) | 62.8(59.1-65.1) | 63.0(59.8-66.3) | 0.45^†^ |
| LV mass (g) | 79.2(71.2-99.6) | 85.5(75.6-110.3) | 0.15^†^ |
| LV mass-Index -BSA (mg/m^2^) | 41.9(37.2-50.1) | 46.7(41.2-53.3) | **0.01**^†^ |
| RVEF (%) | 54.1(49.8-57.6) | 53.3(50.1-58.1) | 0.77* |
| RVEDV (ml) | 154.1(138.4-197.3) | 157.5(134.5-183.7) | 0.96^†^ |
| RVEDV-Index -BSA (ml/m^2^) | 83.2(71.6-94.2) | 84.9(76.0-97.7) | 0.56^†^ |
| RVSV (ml) | 86.5(72.4-105.0) | 83.4(73.4-100.3) | 0.79^†^ |
| RVSV-Index -BSA (ml/m^2^) | 45.9(37.7-52.0) | 45.0(39.9-52.3) | 0.50* |
| LA (cm^2^) | 22.0(18.5-24.3) | 21.1(18.6-23.4) | 0.61^†^ |
| LA EF (%) | 64.3(58.8-67.0) | 62.7(59.2-69.2) | 0.77^†^ |
| LA-EDV-Index-BSA (ml/m) | 35.0(27.8-40.2) | 33.2(28.2-38.7) | 0.67^†^ |
| RA (cm^2^) | 21.0(19.0-25.1) | 21.1(19.5-24.2) | 0.68* |
| RA EF (%) | 49.1(43.8-55.1) | 51.3(46.1-57.7) | 0.37^†^ |
| Global longitudinal strain (%) | -17.8(-18.9-(-15.8) | -17.1(-19.0-(-16.1) | 0.95^†^ |
| Global radial strain (%) | 23.3(21.7-28.3) | 28.0(24.1-31.1) | **0.002*** |
| Global circumferential strain (%) | -15.4(-17.7-(-14.7) | -17.5(-18.6-(-15.9) | **0.002*** |
| T1 global (ms) | 1004(980-1023) | 991(968-1005) | **0.01*** |
| T1 basal (ms) | 1001(982-1024) | 992(970-1010) | **0.02*** |
| T1 midventricular (ms) | 1001(975-1024) | 986(958-1001) | **0.01*** |
| T2 global (ms) | 48(47-50) | 50(48-51) | **0.01*** |
| T2 basal (ms) | 49(47-50) | 50(48-51) | **0.02*** |
| T2 midventricular (ms) | 49(47-50) | 50(48-51) | **0.003*** |

Data provided as absolute and percent or median and interquartile range. LV=left ventricle, EDV=end-diastolic volume, BSA=body surface area, ESV=end-systolic volume, SV=stroke volume, EF=ejection fraction, RV=right ventricle, LA=left atrium, RA=right atrium, ECV=extracellular volume, LGE=late gadolinium enhancement. *T-tests, ^†^Mann-Whitney-U test.
