# Supplementary material for: Deep cardiac phenotyping by cardiovascular magnetic resonance reveals subclinical focal and diffuse myocardial injury in patients with psoriasis (PSOR-COR study)
Source: Clin Res Cardiol. 2024 May 16;114(9):1133–44. doi: 10.1007/s00392-024-02456-9 (PMC12408704; doi:10.1007/s00392-024-02456-9)
Supplement: Supplementary file 8 — Supplementary file8 (DOCX 16 KB) [file 392_2024_2456_MOESM8_ESM.docx]

Supplementary table 6 Cardiac function and tissue parameters for the mild and moderate/severe psoriasis cohort

| Parameter | Mild Psoriasis (mPV) (N=24) | Moderate/Severe Psoriasis (sPV) (N=36) | *p*-value mPV vs. sPV |
| --- | --- | --- | --- |
| LVEDV (ml) | 135.0 (123.7-151.1) | 152.3 (117.0-181.4) | 0.26^†^ |
| LVEDV-Index-height (ml/m) | 79.4 (73.3-87.2) | 85.1 (68.8-100.1) | 0.41^†^ |
| LVEDV-Index -BSA (ml/m^2^) | 71.1 (62.0-80.4) | 74.7 (62.5-85.9) | 0.54^†^ |
| LVESV (ml) | 48.7 (44.1-58.4) | 57.0 (42.3-69.4) | 0.35^†^ |
| LVSV (ml) | 86.5 (78.4-95.8) | 92.9 (74.9-112.2) | 0.25^†^ |
| LVSV-Index -BSA (ml/m^2^) | 43.9 (40.0-50.8) | 46.3 (42.2-53.2) | 0.26^†^ |
| LVEF (%) | 62.7 (59.7-66.7) | 63.2 (58.5-65.6) | 0.91* |
| LV mass (g) | 82.1 (69.6-111.7) | 80.9 (71.5-98.4) | 0.71^†^ |
| LV mass-Index -BSA (mg/m^2^) | 43.1 (37.6-52.5) | 41.3 (37.2-49.3) | 0.47^†^ |
| RVEF (%) | 55.7 (53.0-57.5) | 54.0 (49.4-57.7) | 0.13* |
| RVEDV (ml) | 142.7 (129.7-165.0) | 171.9 (131.6-205.9) | **0.046**^†^ |
| RVEDV-Index -BSA (ml/m^2^) | 75.4 (66.6-85.0) | 82.1 (69.1-99.8) | 0.07^†^ |
| RVSV (ml) | 81.3 (72.3-90.1) | 88.3 (67.2-108.3) | 0.15^†^ |
| RVSV-Index -BSA (ml/m^2^) | 43.0 (35.4-47.8) | 45.8 (37.2-53.0) | 0.19* |
| LA (cm^2^) | 21.7 (19.5-23.5) | 23.0 (18.3-25.9) | 0.89* |
| LA EF (%) | 64.1 (55.1-71.8) | 63.7 (58.1-67.8) | 0.56* |
| LA-EDV-Index-BSA (ml/m) | 34.4 (28.5-42.1) | 34.3 (27.4-41.5) | 0.81^†^ |
| RA (cm^2^) | 20.9 (17.9-24.7) | 21.3 (19.8-25.2) | 0.36* |
| RA EF (%) | 50.6 (44.6-56.5) | 48.7 (41.3 -54.8) | 0.23^†^ |
| Global longitudinal Strain (%) | -17.4 (-18.1-(-14.5)) | 16.8 (-18.4-(-15.7)) | 0.79^†^ |
| Global radial Strain (%) | 23.2 (20.8-26.9) | 24.2 (21.8-28.6) | 0.33* |
| Global circumferential Strain (%) | -15.4 (-16.9-(-14.4)) | -15.8 (-17.7-(-14.7)) | 0.30* |
| T1 global (ms) | 986.8 (974.4-1011.7) | 1006.6 (989.6-1027.5) | 0.19* |
| T1 basal (ms) | 990.1 (977.3-1017.7) | 1006.0 (995.8-1027.1) | 0.24* |
| T1 midventricular (ms) | 989.0 (973.4-1010.9) | 1009.2 (976-1030.4) | 0.20* |
| T2 global (ms) | 48.0 (47.2-48.9) | 48.4 (47.0-49.6) | 0.37* |
| T2 basal (ms) | 48.2 (47.3-49.0) | 48.5 (47.0-50.0) | 0.55* |
| T2 midventricular (ms) | 47.9 (46.7-48.8) | 48.4 (46.8-49.9) | 0.27* |

Data provided as absolute and percent or median and interquartile range. LV=left ventricle, EDV=end-diastolic volume, BSA=body surface area, ESV=end-systolic volume, SV=stroke volume, EF=ejection fraction, RV=right ventricle, LA=left atrium, RA=right atrium, ECV=extracellular volume, LGE=late gadolinium enhancement. *T-tests, ^†^Mann-Whitney-U test, ^‡^Chi-square test or Fisher’s exact test.
